# NAIR: Network Analysis of Immune Repertoire

**DOI:** 10.3389/fimmu.2023.1181825

**Published:** 2023-07-07

**Authors:** Hai Yang, Jason Cham, Brian Patrick Neal, Zenghua Fan, Tao He, Li Zhang

**Affiliations:** ^1^ Helen Diller Family Comprehensive Cancer Center, University of California San Francisco, San Francisco, CA, United States; ^2^ Department of Medicine, Scripps Green Hospital, La Jolla, CA, United States; ^3^ Department of Medicine, University of California San Francisco, San Francisco, CA, United States; ^4^ Department of Mathematics, San Francisco State University, San Francisco, CA, United States; ^5^ Department of Epidemiology and Biostatistics, University of California San Francisco, San Francisco, CA, United States

**Keywords:** adaptive immune response, sequencing generation probability, network analysis, SARS-CoV-2, T cell repertoire sequencing

## Abstract

T cells represent a crucial component of the adaptive immune system and mediate anti-tumoral immunity as well as protection against infections, including respiratory viruses such as SARS-CoV-2. Next-generation sequencing of the T-cell receptors (TCRs) can be used to profile the T-cell repertoire. We developed a customized pipeline for Network Analysis of Immune Repertoire (NAIR) with advanced statistical methods to characterize and investigate changes in the landscape of TCR sequences. We first performed network analysis on the TCR sequence data based on sequence similarity. We then quantified the repertoire network by network properties and correlated it with clinical outcomes of interest. In addition, we identified (1) disease-specific/associated clusters and (2) shared clusters across samples based on our customized search algorithms and assessed their relationship with clinical outcomes such as recovery from COVID-19 infection. Furthermore, to identify disease-specific TCRs, we introduced a new metric that incorporates the clonal generation probability and the clonal abundance by using the Bayes factor to filter out the false positives. TCR-seq data from COVID-19 subjects and healthy donors were used to illustrate that the proposed approach to analyzing the network architecture of the immune repertoire can reveal potential disease-specific TCRs responsible for the immune response to infection.

## 1 Introduction

T cells are a vital component of the adaptive immune system and are responsible for defending against infection. The unique T-cell receptor (TCR) on each T cell dictates antigen specificity. Collectively, all of an individual’s TCRs make up the T-cell immune repertoire. Thus, investigating the immune repertoire is paramount to understanding the basis underlying the immune response to infection (1). Because of the enormous breadth of epitopes recognized by the adaptive immune system, the T-cell immune repertoire is highly diverse and dynamic. Repertoire dynamics span several orders of magnitude in size (germline gene to clonal diversity), physical location (circulation, lymph nodes, and tissues), and time (short-lived responses to immunological memory that can persist for decades) (2–7). Advancements in next-generation sequencing technology have allowed researchers to sequence deeply enough to provide a comprehensive profile of the high-dimensional complexity of the adaptive immune receptor repertoire (AIRR-seq).

Recently, the AIRR-seq analysis has been applied to COVID-19 subjects to understand how the adaptive immune system is induced by SARS-CoV-2 (8). A higher proportion of somatic hypermutation was associated with more severe COVID-19 infection (9, 10). Global analysis of the TCR repertoire in COVID-19 subjects revealed that recovered subjects had increased diversity and richness above healthy individuals and that the VJ gene usage in the TCR beta chain was skewed. Overall, this type of immune repertoire analysis demonstrates the excellent potential to be a biomarker for improved diagnosis and monitoring of disease.

Unlike the immune repertoire diversity, which is based on the frequency profiles of individual clones (11), sequence similarity architecture captures frequency-independent clonal sequence similarity relations. Conserved sequences in the complementarity-determining region 3 (CDR3) region within the immune receptors directly influence the antigen recognition breadth: The more different receptors are, the larger the antigen space covered. Network analysis clusters TCRs based on sequence similarity and thereby adds a complementary layer of information to repertoire diversity analysis. However, only networks with hundred thousand nodes can be visualized, and such visualization of networks only provides marginal quantitation of the network similarity architecture. Graph properties and quantitative analysis of network analysis have been recently employed to quantify the network architecture of immune repertoires (12). These advancements provide better understanding of the fundamental properties of repertoire architecture such as reproducibility, robustness, and redundancy (13).

Public or shared T-cell clones are T cells that have the exact same CDR3 nucleotide or amino acid sequence between individuals or within an individual across time (14). Functionally, public (shared) clones are enriched for Major histocompatibility complex-diverse CDR3 sequences previously associated with autoimmune, allograft, tumor-related, and anti-pathogen–related reactions (15). Public clones from different time points or specimens belonging to the same subject are more likely to be antigen specific (15). However, it is also possible for public clones to target epitopes that are shared with other diseases. Public clone searching can identify common and similar TCRs (defined as a cluster in network analysis) but might miss the rare TCRs closely related to the disease, especially clusters with small sizes. Therefore, we propose customized pipelines to identify the disease-associated clusters to find the rare TCRs closely associated to disease.

Probability of generation (p_gen_) evaluates which specific amino acid sequences and sequence motifs are likely to be generated and found in repertoires (16, 17). It is essential to distinguish the antigen-driven clonotypes from genetically naïve predetermined clones. A higher generation probability of a given receptor sequence leads to a higher chance of finding it in any given individual. Therefore, public or shared clones usually have a higher generation of probability. The probability of generating any nucleotide sequence is defined as the sum of probabilities for all generative events that could potentially produce that sequence (16, 17). Here, we introduced a new metric to evaluate the importance of the clones by incorporating both generation probability and clonal abundance by utilizing Bayes factor.

**Figure 1:**
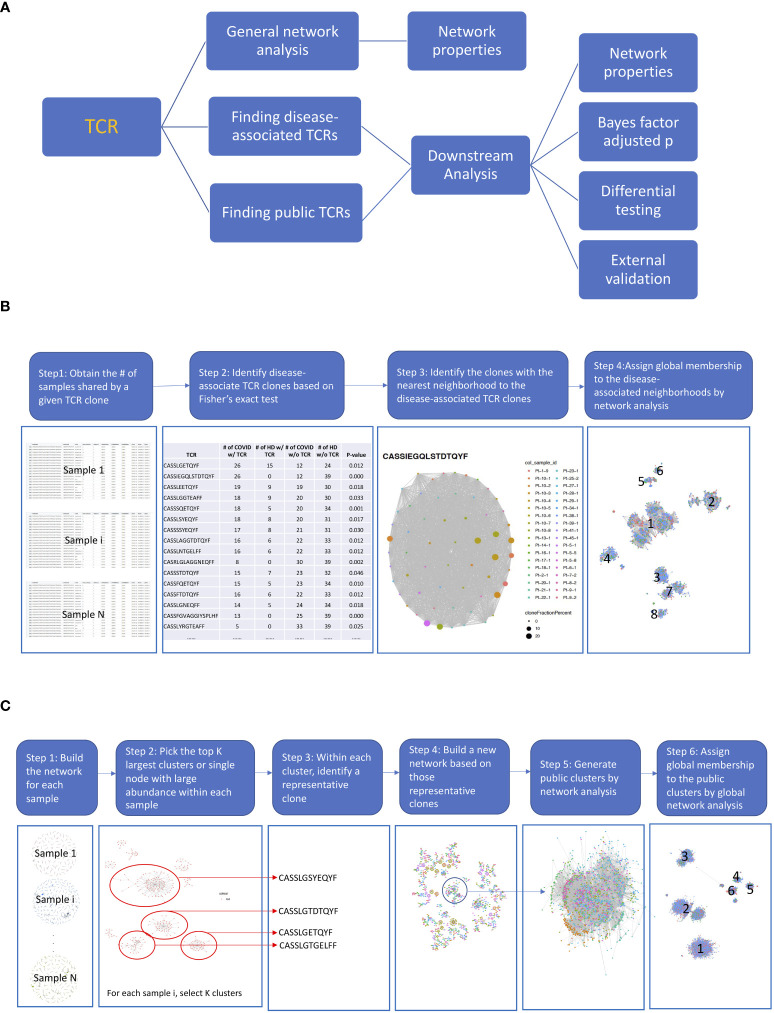
The diagrams of the proposed pipelines. **(A)** Overall roadmap. We started with a general network analysis for each sample and correlated the network properties with the subjects’ clinical characteristics. We then developed pipelines to find disease-associated clusters and shared clusters across samples to identify antigen-driven T-cell receptors (TCRs) with downstream analysis. **(B)** Finding disease-associated clusters pipeline. (1) First, we obtained the number of samples share by a given TCR. (2) Then, we identified the COVID-associated TCRs, based on their presenting frequency in COVID subjects comparing to that of healthy samples using Fisher’s exact test (*p*< 0.05) and shared at least by 10 samples. We only kept the TCRs with length >= 6. (3) For each COVID-associated TCRs, we identified the TCRs that were in the same cluster by searching among all TCRs from shared samples by network analysis (hamming distance<= 1). The TCR clusters now only present in COVID samples were defined as COVID-only TCR clusters and rest were COVID-associated TCR clusters. (4) Last, we generated a network across all COVID-associated TCRs including their member TCRs in the same cluster and assigned global membership to the COVID-associated clusters. **(C)** Finding public clusters workflow. (1) First, we built the network for each sample. (2) Within the network for each sample, we picked the top K largest clusters or the single node with a large abundance (count > 100). (3) Within each cluster, we identified a representative clone with the largest count. (4) We built a new network based on those selected clones, and the clusters with clones from different samples were considered as the skeleton of public clusters. (5) We generated public clusters by expanding each skeleton public cluster to include any clones belonging to the same cluster in the original sample by another network analysis. (6) We assigned global membership to the public clusters based on Step 5.

GLIPH2 (18) and ImmunoMap (19) can also be applied to bulk AIRR-seq data to identify potential targets for immunotherapeutic interventions in various diseases. GLIPH works by clustering TCR sequences based on the similarity of sequences, while ImmunoMap works by using a database of known antigens to identify the antigen specificities of TCR sequences. However, they both lack a more comprehensive searching algorithm (e.g., disease specific) and downstream analysis to related with the clinical characteristics/outcome. Our primary objective in this paper is to develop such comprehensive search algorithms and downstream analysis (**Figure 1A**). We applied the proposed approaches and pipelines to publicly available AIRR-seq data of a group of European COVID-19 subjects and healthy donors (20) to identify COVID-19–specific and COVID-19–associated TCRs, and we validated our findings using the MIRA (Multiplex Identification of Antigen-Specific T-Cell Receptors Assay) database (21).

## 2 Materials and methods

### 2.1 European COVID data

The TCR-sequencing data from the European COVID-19 subjects (20, 22) includes three cohorts: a cohort of subjects who recovered after COVID-19 with mild-to-moderate disease courses (*n* = 19), a cohort of subjects with active infection and severely symptomatic who had comorbidities (*n* = 18), all of which required hospitalization and an age-matched healthy donor cohort tested negative for COVID-19 antibodies (*n* = 39). Up to nine follow-up blood samples were available per subject, some spanning different disease stages in the same subject (e.g., two recovered subjects, Patients 6 and 7, also had one and three samples collected during they were actively infected), totaling 108 samples (**Supplementary Table S1**). The AIRR-seq data include 19 recovered samples from the recovered subjects (additionally, four samples during active infection), 46 samples during active infection from 18 subjects with active infection, and 39 samples from the healthy donors. The characteristics of the subjects were shown in (20) (gateway.ireceptor.org; Study ID: IR-Binder-000001). As described in (20), next-generation sequencing of the TCR beta chain was performed for all acquired blood samples. Each unique CDR3 amino acid sequence was defined as one clone. There were 901,045 unique TCRs. Annotation of TCR loci rearrangements was computed with the MiXCR framework (3.0.13) (23). The default MiXCR library was used for TCR sequences as the reference for sequence alignment. More specifically, we used “analyze shotgun” pipeline with setting –species hsa –starting-material rna. Non-productive reads and sequences with less than two read counts were not considered for further analysis.

### 2.2 Adaptive MIRA database

Adaptive Multiplex Identification of Antigen-Specific T-Cell Receptors Assay (MIRA) was used to identify antigen-specific TCRs (21). The COVID-19 MIRA dataset maps TCRs binding to SARS-Cov-2 virus epitopes and includes data from exposed subjects and naive controls. The COVID-19 MIRA dataset contains more than 135,000 high-confidence SARS-CoV-2–specific TCRs. Data are available at https://clients.adaptivebiotech.com/pub/covid-2020;_DOI 10.21417/ADPT2020COVID.

### 2.3 Network analysis

The pairwise distance matrix of TCR amino acid sequences for each subject was calculated using Hamming distance (Python module SciPy with pdist function). When Hamming distance is less than or equal to 1, then the edge is equal to 1; otherwise, it is equal to 0. A network cluster was defined as a group of clones with a Hamming distance less than or equal to 1 (allowing a maximum of one base pair mismatch among clone sequences) by fast greedy algorithm (igraph (24)). Network visualization was performed using R packages: igraph and ggraph. Each node represents a single TCR amino acid CDR3 sequence. The patterns of the sequences within a cluster were visualized by sequence logos using R package: ggseglogo (25). In addition, we have included network features as one of the major outputs to describe the network. There are two types of network properties (13): global properties which describe the network as whole and local properties which characterize clonal features for each node in repertoire networks (**Supplementary Table S2**). To quantitatively correlate the network with clinical characteristics/outcome, for example, healthy donors *versus* COVID-19 samples coming from multiple time points, we applied a generalized linear mixed model to account for the repeated measures, where we focused on the global properties. Specially, for each global property, we used the maximum value within a given sample as the outcome variable, since we usually have many clusters within one sample. We then treated time (the number of weeks since diagnosed with COVID) and sample characteristics, such as COVID active, COVID received, or healthy, as fixed effects, while the subject was considered as a random effect. This approach allowed us to account for multiple samples from the same subject and to compare the maximum property values across different groups. In addition, for each global property, we used heatmaps to display the distribution of all values for each cluster (columns) across the samples (rows), while the dendrogram on the left side shows the hierarchical clustering based on the corresponding property values.

### 2.4 Analysis pipeline

The disease-associated TCR cluster is characterized as a group comprising TCRs that exhibit, at most, one amino acid difference in their TCR sequences and display a statistically significant difference in their frequency between the disease group and the control group. As illustrated in **Figure 1B**, first, we obtained the number of samples share by a given TCR. To identify the COVID-associated TCRs, we performed a Fisher exact test. Specifically, we calculated the number of samples that shares a given TCR sequence, resulting in a 2 × 2 table. The first row of the table includes two numbers: the number of healthy donors who share and do not share the given TCR sequence. The second row includes two numbers: the number of COVID-19 patients who share and do not share the given TCR sequence. We used Fisher’s exact test (*p*< 0.05) to identify TCR sequences that are potentially associated with COVID-19 based on this 2 × 2 table. To identify clusters of COVID-associated TCRs, we searched for TCRs that were in the same cluster as the COVID-associated TCRs by analyzing all TCRs from shared samples using network analysis (with a Hamming distance of<= 1). The TCR clusters that were found only in COVID-19 samples were defined as COVID-only TCR clusters, while the rest were defined as COVID-associated TCR clusters. Finally, we constructed a network across all COVID-associated TCRs, including their member TCRs in the same cluster, and assigned global membership to the COVID-associated clusters.

We proposed a workflow to identify the public clusters (**Figure 1C**) by a customized search algorithm. The public cluster encompasses TCRs that exhibit a maximum of one amino acid disparity in their TCR sequences across individuals or within an individual over time. Within the network for each sample, we picked the top K largest clusters or a single node with a high abundance (count > 100). Next, we selected a representative clone with the largest count within each cluster. We then built a new network using these selected clones, and the clusters that contained clones from different samples were considered as the skeleton of public clusters. To generate public clusters, we expanded each skeleton public cluster to include any clones that belonged to the same cluster in the original sample by another network analysis. Finally, we assigned global membership to the public clusters based on the previous step.

We also proposed downstream analysis to identify interesting disease-specific and public clusters by testing across sample types. Last, we validated our findings by exactly matching with the TCR clones in MIRA datasets. All analyses, unless noted, were done by the statistical computing software R and the programming language Python.

### 2.5 Downstream analysis within disease-associated clusters and public clusters

#### 2.5.1 Differential testing of the TCR clusters

Once we assigned the global membership based on either Public Clusters or Disease Associated Clusters Pipeline, we treated each global cluster as a feature and then performed differential testing. First, we aggregated the TCR clonal count for each sample for those TCRs belong to the same global cluster. Next, to perform differential testing of the aggregated counts between groups defined by clinical characteristics/outcome, for example, active COVID-19 samples *versus* healthy donors, recovered COVID-19 samples *versus* healthy donors and active COVID-19 samples *versus* recovered COVID-19 samples coming from multiple time points, we applied a generalized linear mixed model to account for the repeated measures. We first aggregated counts for each global cluster within a given sample, normalized the aggregated counts by dividing them by the sample read depth, and then applied a logarithmic transformation to the normalized values, which served as the outcome variable. We treated time and sample characteristics, such as COVID active, COVID received, or healthy, as fixed effects, while accounting for the subject as a random effect. This enabled us to control for multiple samples from the same subject and to compare the cluster aggregated counts across different groups.

#### 2.5.2 Correlation between TCRs based on Atchley factor

To analyze the TCR sequences within a cluster, we first visualized the sequence pattern of each cluster through a sequence logo (25), and then characterized each TCR biochemically using its Atchley factor (26). The overall biochemical properties of any amino acid sequence can be expressed as a sequence of five Atchley factor values, which correspond loosely to polarity, secondary structure, molecular volume, codon diversity, and electrostatic charge (26). For the TCR clones that belong to a TCR cluster, we first used TESSA software (27) to create a numeric embedding of TCRs, where each numeric vector represented a TCR sequence. Then, the pairwise Pearson’s correlation coefficient among the Atchley factor encoded TCRs within a cluster can be calculated, and their median and interquartile range (IQR) can be obtained as a measure of the similarity within the cluster.

#### 2.5.3 Clustering samples based on TCRs

We first quantified the number of TCRs belonging to each TCR cluster (defined based on network analysis as either COVID-associated or public) in each sample and then normalized this value by dividing it by the total number of TCRs within the sample. Next, we calculated the correlation coefficient based on the normalized number of TCRs in each TCR cluster across all samples. We clustered the samples by hierarchical clustering based on the normalized frequencies calculated in the previous step.

#### 2.5.4 Probability of generation and Bayes factor adjusted *P*-value (false discovery rate)

We introduced a new metric to evaluate the importance of the clones by incorporating clonal generation probability (*p*_gen_) and clonal abundance using Bayes factor to evaluate the significance of identified clones. We then calculated Bayes factor adjusted *p*-value and false discovery rate (FDR) for each TCR and summarized the proportion of the TCRs with Bayes adjusted *FDR*< 0.05.

The Bayes factor is the ratio of two marginal likelihoods. Clonal generation probability (*p*_gen_) probabilistically annotates sequences, and its modular structure can be used to investigate models of increasing biological complexity for different organisms, which is calculated by OLGA (28). For each clone $M_{c}$ , Bayes factor between clone $M_{c}$ and clone $M_{j}$ is calculated by


$$BF_{c}(j))=\frac{P\left(M_{c}|D\right)/P(M_{c})}{P\left(M_{j}|D\right)/P\left(M_{j}\right)}\text{ for }c\neq j\text{ and }c,\;j=1,\;\ldots ,K,$$

where $P\left(M_{j}\right)\;$ is the *p*_gen_ of clone $M_{j}$ , and $P\left(M_{j}|D\right)\;$ is the normalized frequencies of clone $M_{j}\;$ in the repertoire D , j=1, …,K . Thus, each clone $M_{c}$ has a vector of K−1 of $BF_{c}\left(j\right)$ values corresponding to K−1 clones in the same repertoire. We are interested in the proportion of X = log_10_( $BF_{c}\left(j\right)$ ) $\geq x_{0}$ because log_10_( $BF_{c}\left(j\right)$ ) falls between the intervals of (0.5, 1), (1, 2), and > 2, representing substantial, strong, and decisive chance presented in the current data, respectively (29). Here, we can consider $x_{0}=2$ . Note that, under the null hypothesis, X follows a normal distribution with a mean of 0 and a standard deviation of σ ( σ will be estimated through the real data). Let Z be the number of log_10_( $BF_{c}\left(j\right)$ ) $\geq x_{0}\;$ , for c≠j and c, j=1, …,K , then under the null hypothesis, Z follows a binomial distribution P(Z|p)~ Binomial (K−1, p) with a probability of $p\;=\int_{x_{0}}^{\infty }f\left(x\right)dx$ , where f(x) is a normal density function. Then, we can calculate a pseudo *p*-value $P_{BF}^{c}$ = $\sum_{Z\geq z_{0}}P(Z|p)$ for each clone c , corresponding to the probability that clone c has no less than $z_{0}$ of $BF_{c}\left(j\right)\geq x_{0}\;$ in the null model than in the data. $z_{0}$ can be calculated within each sample as the total of the clones which have log_10_( $BF_{c}\left(j\right)$ ) $\geq x_{0}$ . Those clones with $FDR_{BF}^{c}$ < 0.05 will have a high potential to be COVID-specific TCRs, where $FDR_{BF}^{c}$ is adjusted $P_{BF}^{c}$ (30).

#### 2.5.5 MIRA validation

We first conducted an exact matching to identify TCR sequences found in the European COVID-19 dataset that were exactly the same as those in the MIRA database. We then counted the total number of matching TCR sequences within each cluster and calculated the percentage by dividing this number by the total number of TCRs within that cluster. It allowed us to identify and quantify the degree of overlap between the TCR sequences found in the European COVID-19 dataset and those in the MIRA database and provided insights into the potential relevance of these sequences to COVID-19 immunology.

## 3 Results

### 3.1 TCR repertoire responses in SARS-CoV-2

**Figure 2:**
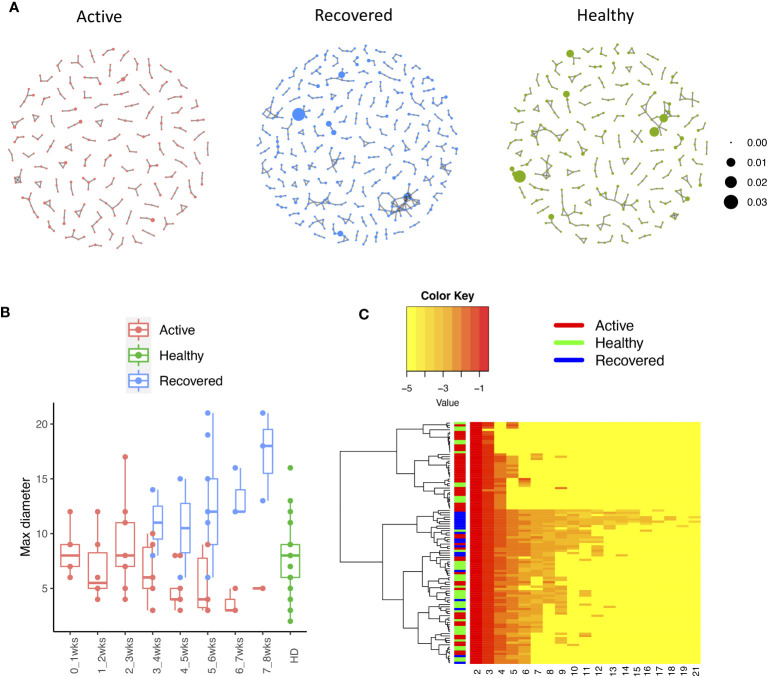
The relationship between the TCR repertoires and sample disease status. **(A)** The network for the representative samples (red: COVID active sample, green: healthy donors, and blue: COVID recovered samples). Each dot represents a single T-cell receptor (TCR), which are connected based on their similarity. The node size is proportional to the TCR clonal abundance. **(B)** Boxplot of the maximum diameter among the clusters for each sample across time by disease status. **(C)** Heatmap of all diameter values for each cluster across samples. Each row represents an individual sample (with left bar presenting the sample information) and each column is the diameter value for each sample, while the dendrogram on the left side shows the hierarchical clustering based on diameter values.

The network analysis (**Figure 2A**, **Supplementary Table S3**) showed that, in the recovered samples, there were more clusters (**Supplementary Figures S1A, B**) identified with larger cluster size (**Supplementary Figures S1C, D**), diameters (**Figures 2B, C**), and assortativity (**Supplementary Figures S1E, F**) compared with healthy donors and active samples (samples collected during active infection), respectively. Interestingly, the repertoire network of active samples had similar cluster size, maximum cluster size, maximum diameter, and maximum assortativity as healthy donors. Additionally, the maximum diameter for active samples went down over the time while that of recovered samples went up. This indicates that the recovered samples tend to have more COVID-specific TCR clones than active samples and healthy donors. This is consistent with a previous study that demonstrated that patients who have recovered from COVID-19 had a more diverse repertoire compared with active COVID-19 infection and healthy donors (20). Perhaps patients with active COVID-19 infection have not developed an adequate T-cell response to clear infection.

### 3.2 Network analysis identifies the COVID-associated clusters

We identified 135 clusters (a total of 10,416 TCRs) associated with the COVID-19 samples based on Fisher’s exact test *p*< 0.05. There are 30 COVID-only clusters, each shared by at least five unique COVID samples (**Table 1**, **Supplementary Table S4**, **Supplementary Figure S2**). Those clusters have a relatively small size (a smaller number of TCRs), and their median *p*_gen_ ranges from 1.2e-07 to 1.6e-17. Although the median *p*_gen_ across the samples in this study is 2e-10, there are 11 COVID-only clusters with median *p*_gen_ less than 2e-10 and some even as low as 1.6e-17, indicating those clusters might be the interesting COVID-specific TCRs. The local network property, coreness, is very close to the number of unique TCRs in the corresponding clusters (**Table 1**). The median correlation coefficients of Atchley factor decoded TCR sequences within each cluster ranges from 0.76 to 1, indicating that TCR sequences within the same cluster are highly similar. These results suggest that some clusters possess almost identical TCR sequences. Among the 30 COVID-only TCR clusters, 17 clusters exhibit a high degree of similarity in their TCR sequences, with only one or fewer sequence variations within each cluster.

**Table 1:** Summary of COVID-only clusters.

Cluster ID^i^	No. of TCRs	Motif^ii^	No. of active COVID samples^iii^	No. of recovered COVID samples^iv^	Recovered vs. active estimate (95% CI) *P* value^v^	Coreness^vi^ median [min, max]	*P*gen^vii^ median [min, max]	Correlation of Atchley factor^viii^ median [IQR]	The % of TCRs matched with MIRA^ix^
4	57	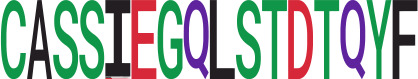	25	11	−1.03(−2.08, 0.03) *p* = 0.058	56[56,56]	2.0e−10 [2.0e−10,5.0e−10]	1[1,1]	0.0%
6	20	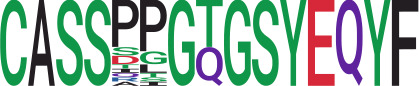	1	9	NA	5[4,6]	8.0e−08 [1.2e−08,3.1e−07]	0.76[0.65,0.85]	40.0%
7	74	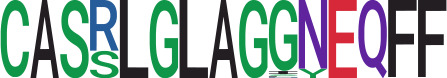	16	2	0.3(−0.26,0.87) *p* = 0.293	55[30,55]	1.4e−08 [1.8e−09,2.5e−07]	0.85[0.76,1]	35.1%
8	31	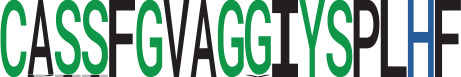	21	5	0.48(−0.05,1.02) *p* = 0.077	27[27,27]	3.9e−13 [1.5e−19,3.9e-13]	1[1,1]	0.0%
10	31	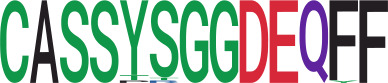	18	4	−0.76 (−2.57,1.06) *p* = 0.413	27[25,27]	7.9e−08 [3.2e−08,1.3e−07]	1[0.98,1]	0.0%
11	25	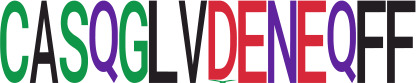	20	4	−1.28 (−3,0.44) *p* = 0.144	24[24,24]	1.7e−12 [1.0e−12,1.7e−12]	1[1,1]	0.0%
12	24	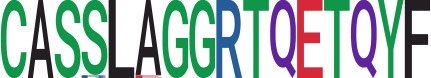	20	2	−0.53 (−2.63,1.57) *p* = 0.623	23[23,23]	5.5e−09 [1.7e−09,5.5e−09]	1[1,1]	0.0%
13	25	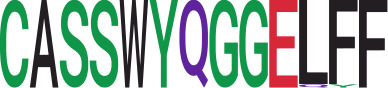	20	3	−1.28 (−3.33,0.76) *p* = 0.218	23[23,23]	4.9e−10 [2.9e−13,4.9e−10]	1[1,1]	0.0%
20	19	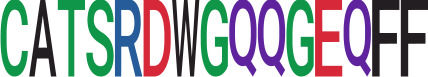	16	3	−1.56 (−3.35,0.23) *p* = 0.088	19[19,19]	1.3e−12 [1.3e−12,1.3e−12]	1[1,1]	0.0%
22	18	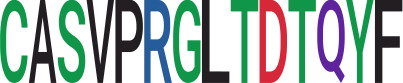	15	3	−0.95 (−2.75, 0.85) *p* = 0.3	18[18,18]	9.3e−10 [9.3e−10,9.3e−10]	1[1,1]	0.0%
23	19	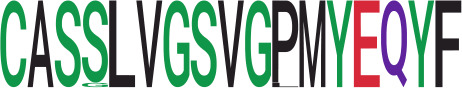	10	6	−0.83 (−1.37, −0.29) *p* = 0.002	17[17,17]	2.2e−13 [5.0e−14,2.2e−13]	1[1,1]	0.0%
25	18	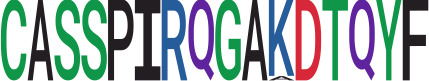	17	0	NA	17[17,17]	2.8e−11 [2.8e−11,1.6e−10]	1[1,1]	0.0%
29	25	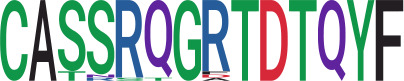	13	2	−0.92 (−2.8,0.96) *p* = 0.339	17[15,17]	1.6e−07 [7.0e−09,1.6e−07]	0.88[0.84,1]	4.0%
30	18	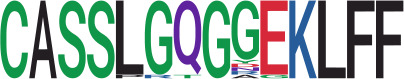	5	3	0.25 (−0.44,0.94) *p* = 0.474	16[12,16]	2.4e−07 [8.1e−08,5.0e−07]	0.915[0.77,1]	11.1%
31	16	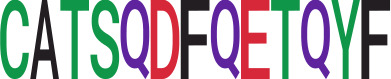	15	1	−1.65 (−4.79,1.49) *p* = 0.303	15[15,15]	3.0e−10 [3.0e−10,3.0e−10]	1[1,1]	0.0%
32	15	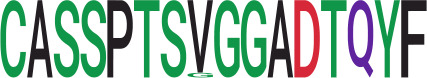	13	1	−1.14 (−4.86,2.57) *p* = 0.546	15[15,15]	9.4e−11 [9.4e−11,3.9e−09]	1[1,1]	0.0%
33	16	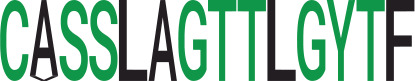	13	2	−1.02 (−3.21,1.18) *p* = 0.363	15[15,15]	8.6e−10 [8.5e−16,8.6e−10]	1[1,1]	0.0%
34	23	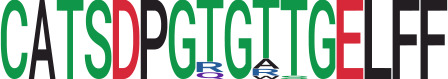	6	1	0.06 (−0.1,0.22) *p* = 0.47	16[11,16]	6.2e−10 [1.0e−10,1.1e−09]	0.96[0.92,0.99]	0.0%
36	33	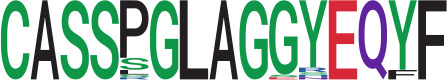	7	3	−0.58 (−2.44,1.29) *p* = 0.544	21[14,21]	1.8e−07 [1.7e−08,4.2e−07]	0.93[0.82,0.98]	75.8%
40	14	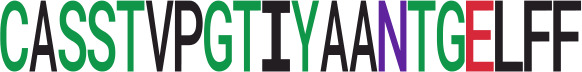	12	2	−0.91 (−3.26,1.44) *p* = 0.448	13[13,13]	1.0e−15 [1.0e−15,1.0e−15]	1[1,1]	0.0%
42	14	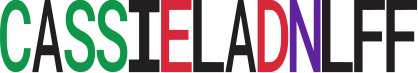	14	0	NA	13[13,13]	7.3e−14 [7.3e−14,7.3e−14]	1[1,1]	0.0%
43	38	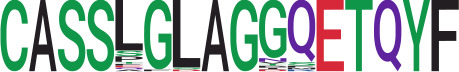	5	5	0.16 (−0.25,0.57) *p* = 0.435	22[12,22]	5.1e−08 [1.1e−09,1.6e−07]	0.9[0.86,0.95]	13.2%
45	13	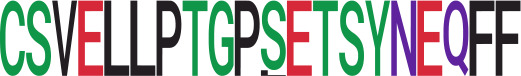	10	2	−1.55 (−4.16,1.07) *p* = 0.246	12[12,12]	1.6e−17 [1.6e−17,2.1e−17]	1[1,1]	0.0%
47	12	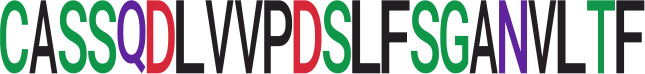	10	2	−2.22 (−3.99, −0.46) *p* = 0.014	11[11,11]	1.2e−15 [1.2e−15,1.2e−15]	1[1,1]	0.0%
49	12	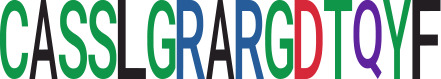	11	1	−1.68 (−5.26,1.89) *p* = 0.356	11[11,11]	2.6e−09 [2.6e−09,2.6e−09]	1[1,1]	0.0%
50	12	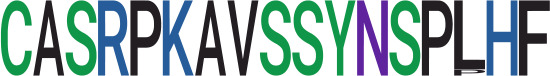	10	1	−0.81 (−2.67,1.06) *p* = 0.397	11[11,11]	4.9e−11 [3.1e−22,4.9e−11]	1[1,1]	0.0%
51	11	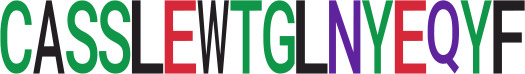	10	1	−1.64 (−4.41,1.13) *p* = 0.245	10[10,10]	1.8e−10 [1.8e−10,1.8e−10]	1[1,1]	0.0%
54	24	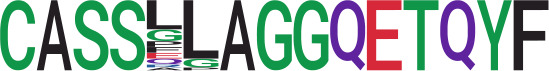	3	5	−0.44 −0.89,0.01) *p* = 0.053	19[9,19]	1.6e−07 [3.9e−08,2.3e−07]	0.87[0.81,0.93]	58.3%
74	11	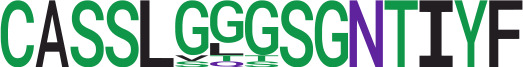	1	6	NA	6[4,6]	2.7e−07 [7.4e−08,5.1e−07]	0.78[0.69,0.89]	18.2%
88	5	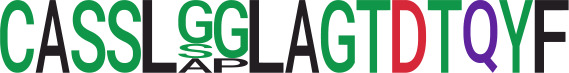	2	3	−0.33 (−1.49,0.83) *p* = 0.58	3[2,3]	1.2e−07 [2.1e−08,1.4e−07]	0.89[0.86,0.92]	0.0%

i Cluster ID is defined based on the network analysis across all samples.

^ii^Sequence logo visualizes all TCR sequences within the corresponding cluster.

^iii^The number of the active COVID samples which the corresponding cluster belongs to.

^iv^The number of the recovered COVID samples which the corresponding cluster belongs to.

v Estimate (with 95% CI) and p-value were obtained based on a linear mixed model or linear model.

^vi^Summary of the coreness (local property) of the TRCs in the corresponding network cluster.

^vii^Summary statistics [median Interquartile range (IQR)] of pgen of TCRs in the corresponding network cluster.

^viii^Summary statistics [median [Interquartile range (IQR)] of pairwise correlation coefficients between the TCR sequences within the public cluster, where the amino acid sequences were transformed by Atchley factor.

^ix^The percentage of the TCRs in the public cluster matched with MIRA.

Clusters 23 and 47, highlighted in light green, had negative estimates and p< 0.05 were considered to be associated with COVID-active samples.

**Figure 3:**
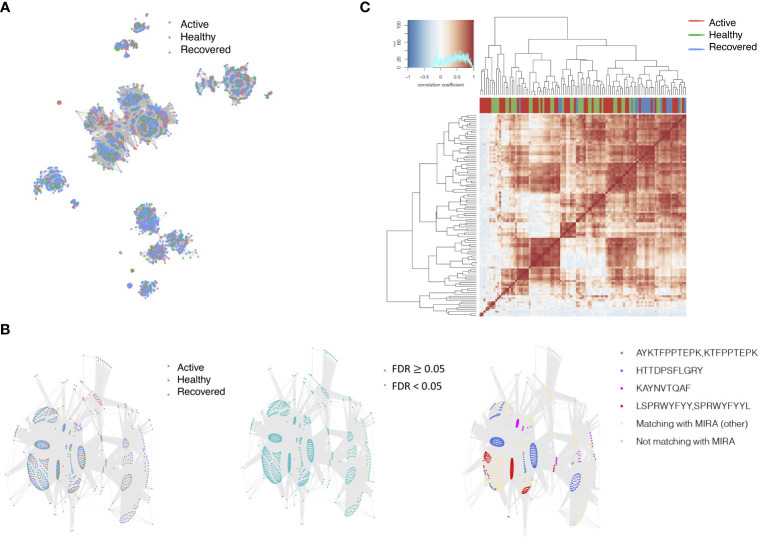
COVID-associated TCR clusters. **(A)** The network of the COVID-associated T-cell receptors (TCRs) whose clusters have statistically significant abundance across subjects’ disease status. Each node represents a single TCR, which are connected if the distance between the two nodes is <= 1 with color coded for the disease status. **(B)** A representative COVID-associated TCR cluster. The plot exhibits network of all TCRs within the selected cluster, where each TCR is color coded based on different metadata information. The right panel presents the corresponding sample’s status, such as active COVID sample, healthy donor, or recovered COVID sample, and the middle panel shows whether the Bayes factor corrected FDR is less than 0.05 or not. The final panel included information on exact matching with MIRA, indicating whether the TCRs match with those identified in the MIRA dataset that bind to specific epitopes. **(C)** Heatmap of pairwise correlation coefficients across samples. The hierarchical clustering of the samples was performed using the Pearson’s correlation coefficient. Correlation coefficient was calculated based on the normalized number of TCRs in each COVID-associated cluster within the samples.

Among the 105 non-COVID-only clusters, we found eight clusters associated with COVID-19 samples (Clusters 1, 3, 9, 14, 48, 58, and 68) and three associated with HD (Clusters 37, 64, and 76) based on differential testing (**Table 2**, **Figures 3A, B**). However, the cluster size varies. Unlike the COVID-only clusters, coreness is relatively smaller than the corresponding cluster size, indicating the increased variability of TCR sequences within the clusters. We found that, for all clusters except Cluster 14, the percentage of significant TCRs based on Bayes factor adjusted FDR ( $FDR_{\mathrm{BF}}$ < 0.05) was higher than 83%, indicating substantial TCRs in these clusters presented in the current data with strong evidence. In addition, most clusters each have at least 50% of TCRs matched with the MIRA dataset, suggesting that TCRs in these clusters have a high potential to be SARS-CoV-2 antigen-specific TCRs. The median of the correlation coefficients of the Atchley factor decoded TCRs within each cluster ranges from 0.51 to 0.86, indicating that all clusters have highly correlated structures. **Figure 3C** presents the sample classification based on correlation coefficient of TCRs.

**Table 2:** Summary of COVID-associated clusters.

Cluster ID^ix^	No. of TCRs	Motif^ix^	No. of HD samples^ix^	No. of active COVID samples^ix^	No. of Recovered COVID samples^ix^	Estimate (95% CI) *P*-value^ix^			Coreness^ix^ Median [Min, Max]	% of significant TCRs based on Bayes factor^ix^	Correlation of Atchley factor^ix^ median [IQR]	The % of TCRs matched with MIRA^ix^
						Active *vs.* HD	Recovered *vs.* HD	Recovered *vs.* active				
1	884	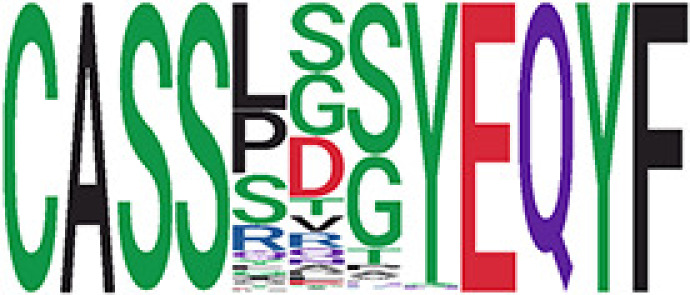	22	32	19	−0.09 (−0.4, 0.23) *p* = 0.588	0.44 (0.08, 0.8) *p* = 0.016	0.53 (0.17, 0.88) *p* = 0.004	146[1, 155]	99.2%	0.55[0.36, 0.74]	82.6%
3	641	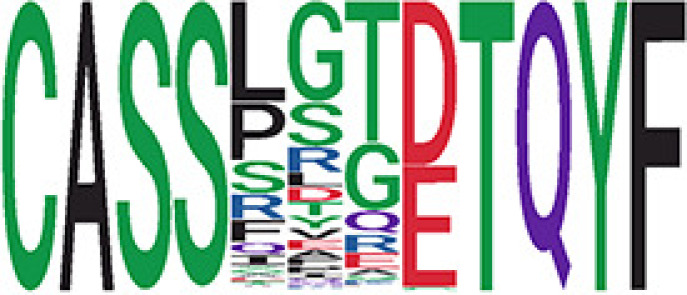	26	32	19	0.33 (−0.09, 0.75) *p* = 0.121	0.66 (0.19, 1.12) *p* = 0.006	0.32 (−0.2, 0.83) *p* = 0.224	75[1,118]	98.1%	0.53[0.39,0.69]	59.6%
9	1668	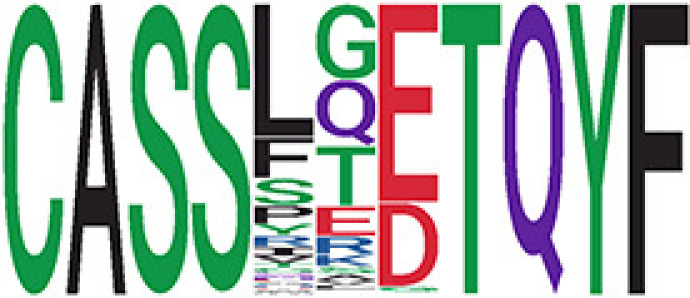	30	34	19	0.26 (0.09, 0.43) *p* = 0.003	0.62 (0.43, 0.82) *p*< 0.001	0.36 (0.18, 0.54) *p*< 0.001	375[10,609]	99.5%	0.67[0.53,0.78]	74.6%
14	24	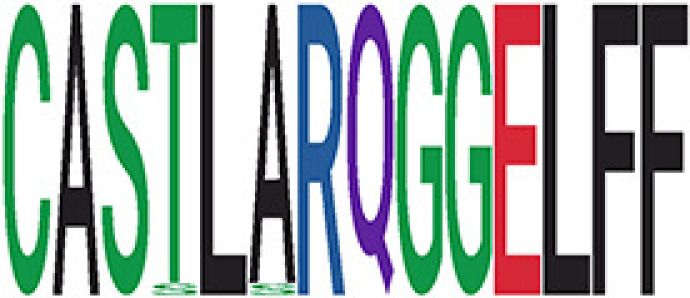	2	14	5	2.24 (1.16, 3.32) *p*< 0. 001	1.63 (0.48, 2.79) *p* = 0.006	−0.6 (−1.39, 0.18) *p* = 0.132	22[22,22]	4.2%	1[1,1]	0.0%
19	71	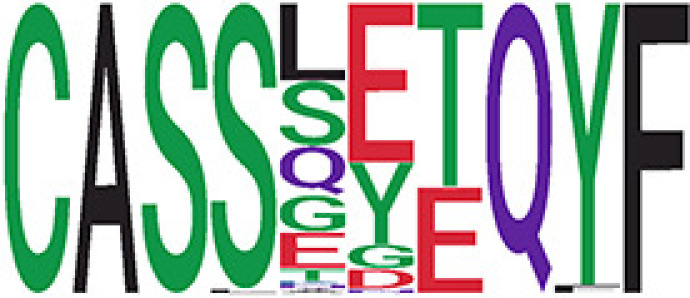	11	10	9	-0.28 (−0.7, 0.14) *p* = 0.186	0.08 (−0.34, 0.5) *p* = 0.712	0.36 (0.11, 0.61) *p* = 0.005	37[1,37]	100.0%	0.59[0.33,0.81]	59.2%
21	317	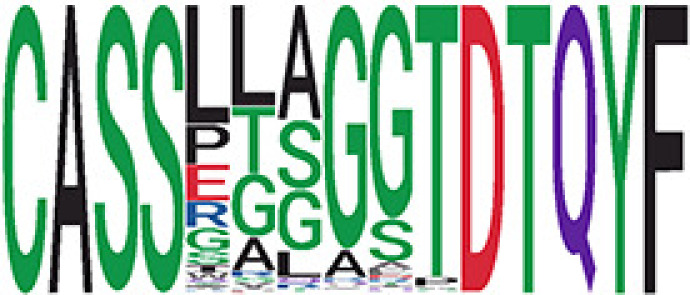	18	22	17	−0.33 (−0.72, 0.06) *p* = 0.101	0.03 (−0.38, 0.45) *p* = 0.883	0.36 (0.02, 0.7) *p* = 0.04	25[1,86]	98.4%	0.61[0.49,0.74]	58.0%
26	224	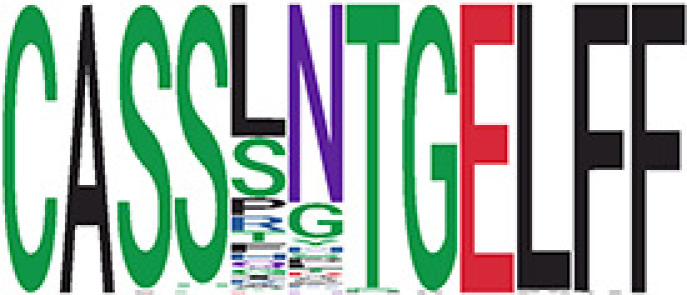	13	15	15	−0.33 (−0.8, 0.15) *p* = 0.178	0.15 (−0.33, 0.62) *p* = 0.54	0.48 (0.01, 0.94) *p* = 0.045	142[1,142]	99.6%	0.78[0.66,0.87]	81.3%
37	18	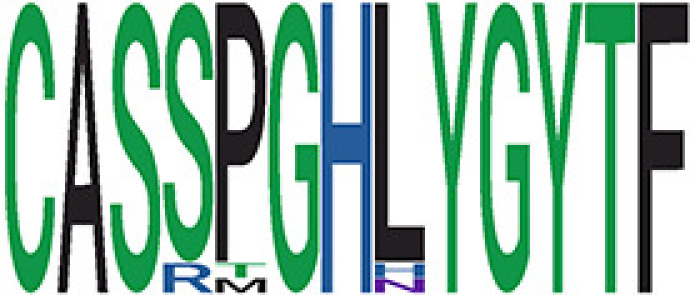	2	8	1	−2.62 (−3.75, −1.5) *p*< 0.001	−3.61 (-5.33, −1.89) *p*< 0.001	−0.99 (−2.59, 0.62) *p* = 0.227	14[14,14]	83.3%	0.86[0.78,1]	0.0%
38	38	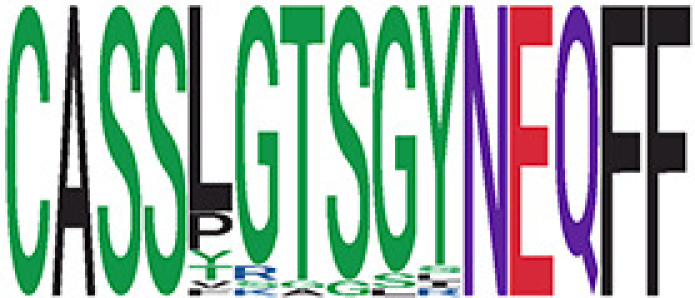	1	2	8	1.04 (0.25, 1.84) *p* = 0.01	0.62 (−0.07, 1.31) *p* = 0.078	−0.42 (−0.94, 0.09) *p* = 0.105	22[13,22]	100.0%	0.82[0.7,0.88]	65.8%
48	933	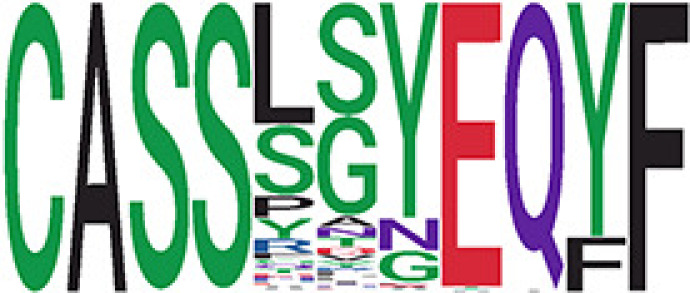	22	32	18	0.09 (−0.21, 0.39) *p* = 0.547	0.5 (0.16, 0.83) *p* = 0.004	0.4 (0.05, 0.75) *p* = 0.024	224[11,343]	99.2%	0.61[0.47,0.76]	88.0%
53	1134	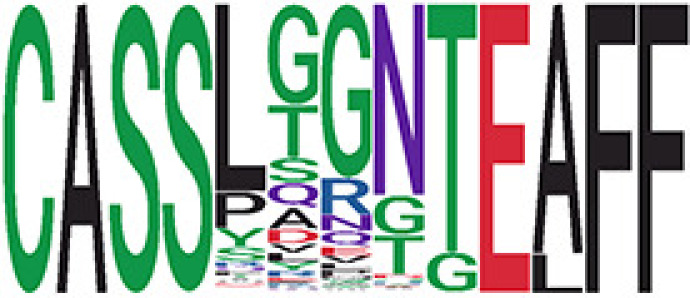	20	37	18	−0.06 (−0.29, 0.17) *p* = 0.612	0.17 (−0.07, 0.41) *p* = 0.156	0.23 (0.01, 0.46) *p* = 0.042	77[4,268]	99.3%	0.58[0.41,0.72]	74.0%
58	91	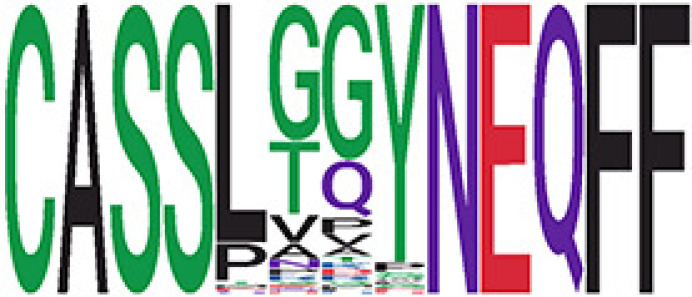	3	7	12	1.11 (0.74, 1.48) *p* =< 0.001	1.1 (0.75, 1.44) *p*< 0.001	−0.01 (−0.28, 0.25) *p* = 0.914	31[4,33]	98.9%	0.66[0.47,0.83]	75.8%
63	8	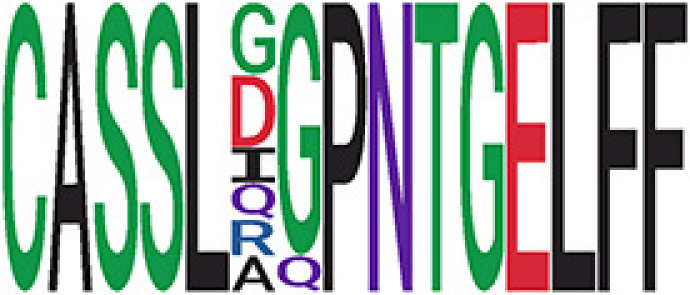	1	1	5	0.14 (−0.84, 1.11) *p* = 0.782	−0.63 (−1.38, 0.13) *p* = 0.103	−0.77 (−1.52, −0.01) *p* = 0.047	6[1,6]	100.0%	0.8[0.74,0.87]	12.5%
64	281	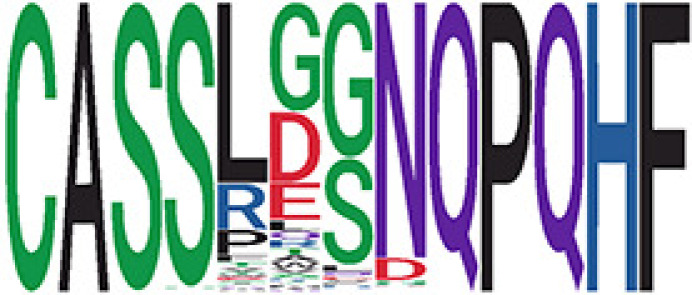	12	18	16	−0.49 (−0.91, −0.07) *p* = 0.022	−0.12 (−0.53, 0.3) *p* = 0.581	0.38 (−0.02, 0.79) *p* = 0.063	64[2,80]	100.0%	0.51[0.36,0.7]	56.9%
68	632	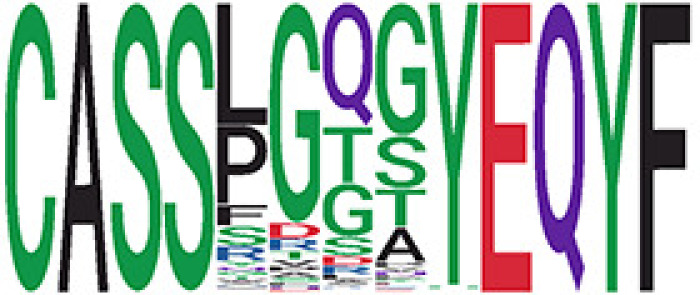	21	25	18	−0.13 (−0.53, 0.28) *p* = 0.543	0.56 (0.11, 1) *p* = 0.014	0.68 (0.25, 1.12) *p* = 0.002	53[1,169]	99.5%	0.59[0.43,0.75]	75.3%
76	11	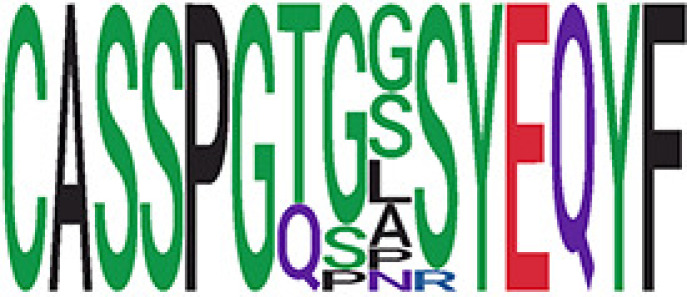	2	1	6	−0.72 (−1.3, −0.13) *p* = 0.017	−0.07 (−0.47, 0.32) *p* = 0.71	0.64 (0.14, 1.14) *p* = 0.012	3[3,4]	100.0%	0.72[0.61,0.87]	72.7%

^ix^Cluster ID is defined based on the network analysis across all samples.

^ix^Sequence logo visualizes TCR sequences within the corresponding cluster.

^ix^The number of the healthy donor (HD) samples which the corresponding cluster belongs to.

^ix^The number of the active COVID samples which the corresponding cluster belongs to.

^ix^The number of the recovered COVID samples which the corresponding cluster belongs to.

^ix^Estimate (with 95% CI) and p-value were obtained based on a linear mixed model or a linear model.

^ix^Summary of the coreness (local property) of the TRCs in the corresponding network cluster.

^ix^The percentage of the significant TCRs within each cluster based on Bayes factor FDR< 0.05.

^ix^Summary statistics [median (interquartile range, IQR)] of pairwise correlation coefficients between the TCR sequences within the cluster, where the amino acid sequences were transformed by Atchley factor.

^ix^The percentage of the TCRs in the cluster matched with MIRA.

Clusters 1, 3, 9, 14, 38, 48, 58, and 68, highlighted in light pink, with positive estimates and p values< 0.05 in either the active versus HD or recovered versus HD columns were considered to be associated with COVID-19 samples. Clusters 37, 64, and 76, highlighted in light green, with negative estimates and p< 0.05 in either the active versus HD or Recovered versus HD columns, were considered to be associated with HD samples. Similarly, Clusters 1, 9, 19, 21, 26, 48, 53, 68, and 76, highlighted in light pink, with positive estimates and p< 0.05 in recovered versus active columns were therefore considered to be associated with COVID-19-recovered samples, while Cluster 63, highlighted in light green, had negative estimates and p< 0.05 was considered to be associated with COVID-active samples.

### 3.3 Network analysis identifies the public clusters shared by different samples and subjects

**Figure 4:**
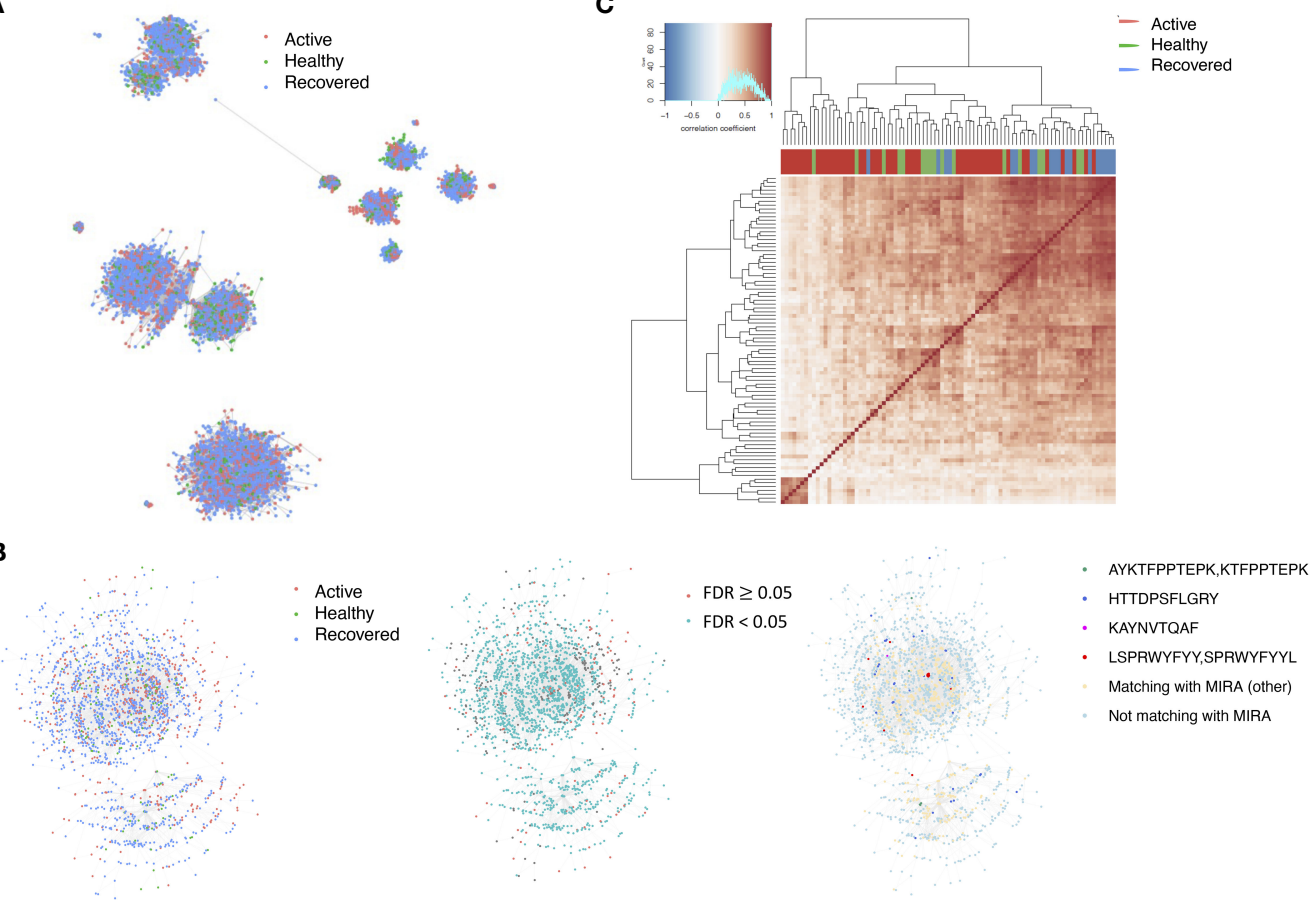
Public shared TCR clusters. **(A)** The network of the public T-cell receptors (TCRs) whose clusters have statistically significant abundance across subjects’ disease status. Each node represents a single TCR, which are connected if the distance between the two nodes is <= 1 with color coded for the disease status. **(B)** A representative public TCR cluster. The plot exhibits network of all TCRs within the selected cluster, where each TCR is color coded based on different metadata information. The right panel presents the corresponding sample’s status, such as active COVID sample, healthy donor, or recovered COVID sample, and the middle panel shows whether the Bayes factor corrected FDR is less than 0.05 or not. The final panel included information on exact matching with MIRA, indicating whether the TCRs match with those identified in the MIRA dataset that bind to specific epitopes. **(C)** Heatmap of pairwise correlation coefficients across samples. The hierarchical clustering of the samples was performed using the Pearson’s correlation coefficient. Correlation coefficient was calculated based on the normalized number of TCRs in each public cluster within the samples.

We identified 1,594 public clusters shared among at least two samples, among which more than two unique individuals shared 170 clusters. Fourteen public clusters were identified by comparing the aggregated clonal abundance across the three groups (healthy donor samples *vs.* COVID active and recovered samples, respectively) (**Table 3**, **Figures 4A, B**). These public clusters usually have huge cluster sizes, and the TCRs in most public clusters have very small coreness, implying relatively high variability among the TCR sequences within each cluster. The median of the correlation coefficients of Atchley factor coded TCRs within each cluster ranges from 0.35 to 0.67, indicating the structures of the clusters are moderately correlated. **Figure 4C** presents the sample classification based on correlation coefficient of TCRs. In addition, we found that except for Clusters 32 and 44, the percent of the significant TCRs ( $FDR_{\mathrm{BF}}$ < 0.05) was higher than 84%, indicating substantial TCRs in these public clusters presented in the current data with a strong chance. Furthermore, three clusters have more than 50% of their TCRS matched with the MIRA dataset. Those results suggested that those TCRs in these public clusters have a high potential to be SARS-CoV-2 antigen-specific TCRs.

**Table 3:** Summary of public clusters.

Public cluster ID^ix^	No. of TCRs	Motif^ix^	No. of HD samples^ix^	No. of active COVID samples^ix^	No. of recovered COVID samples^ix^	Estimate (95% CI) *P*-value^ix^			Coreness^ix^ Median [Min,Max]	The % of significant TCRs based on Bayes factor^ix^	Correlation of Atchley factor^ix^ median [IQR]	The % of TCRs matched with MIRA^ix^
						Active COVID *vs.* HD	Recovered COVID *vs.* HD	Recovered COVID *vs.* active COVID				
1	2092	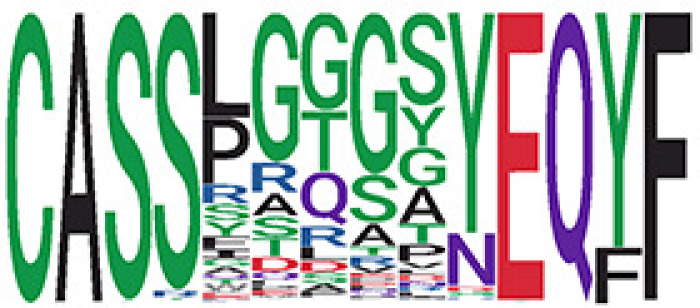	12	39	19	0.33 (0.02, 0.64) *p* = 0.039	0.7 (0.38, 1.02) *p*< 0.001	0.37 (0.11, 0.63) *p* = 0.005	1[1,6]	84.6%	0.37 [0.2,0.53]	28.7%
2	1918	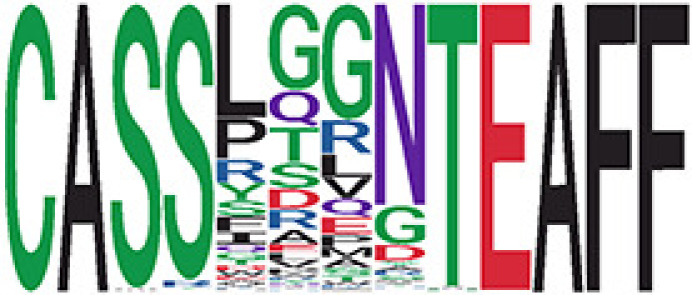	13	40	18	−0.79 (−1.07, -0.51) *p*< 0.001	−0.49 (−0.79, -0.19) *p* = 0.001	0.3 (0.06, 0.54) *p* = 0.015	2[1,7]	95.3%	0.44 [0.28,0.59]	41.6%
4	2321	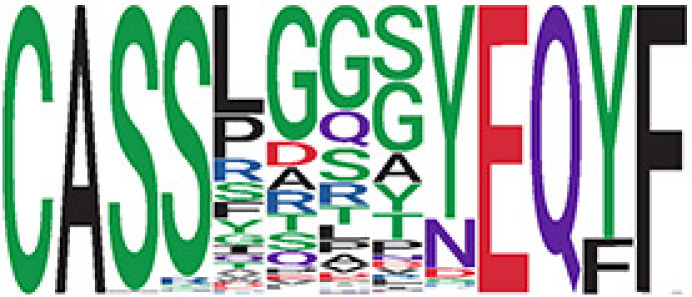	13	40	18	0.46 (0.09, 0.84) *p* = 0.016	0.67 (0.25, 1.09) *p* = 0.002	0.2 (−0.14, 0.54) *p* = 0.24	1[1,6]	90.4%	0.5 [0.34,0.65]	39.5%
6	1585	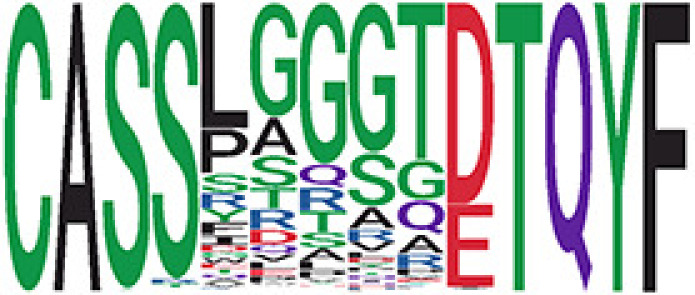	12	39	18	0.41 (0.02, 0.81) *p* = 0.041	0.55 (0.11, 1) *p* = 0.014	0.14(−0.2, 0.48) *p* = 0.424	1[1,4]	86.1%	0.67 [0.55,0.78]	22.1%
7	1011	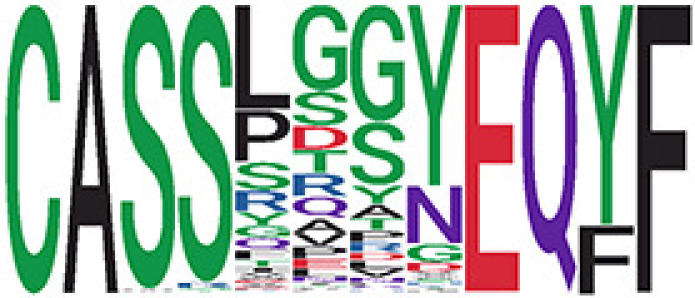	12	38	18	0.38 (0.08, 0.67) *p* = 0.012	0.5 (0.19, 0.82) *p* = 0.002	0.12 (−0.13, 0.38) *p* = 0.342	1[1,8]	90.4%	0.44 [0.28,0.6]	80.3%
8	21799	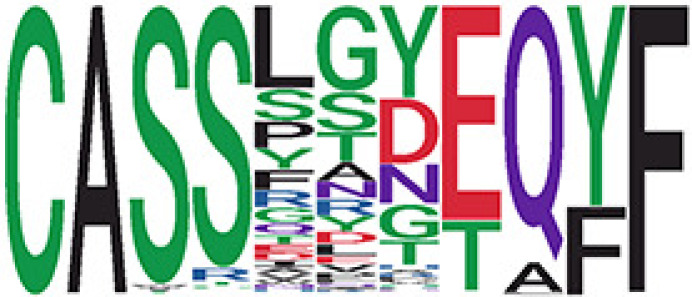	17	39	19	0.25 (−0.02, 0.51) *p* = 0.067	0.46 (0.18, 0.73) *p* = 0.001	0.21 (−0.04, 0.46) *p* = 0.095	2[1,9]	90.5%	0.43 [0.27,0.59]	50.5%
9	782	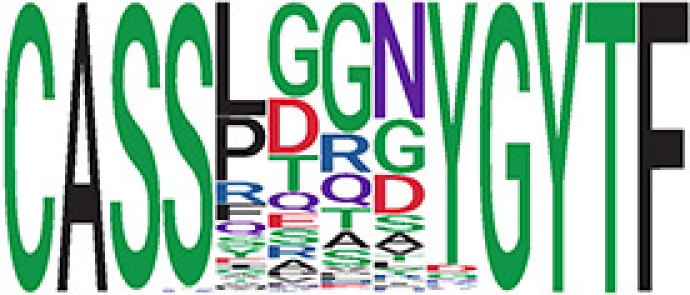	8	24	18	−0.82 (−1.27, −0.38) *p*< 0.001	−0.63 (−1.07, -0.19) *p* = 0.005	0.19 (-0.14, 0.53) *p* = 0.26	1[1,6]	93.4%	0.54 [0.4,0.68]	26.3%
11	894	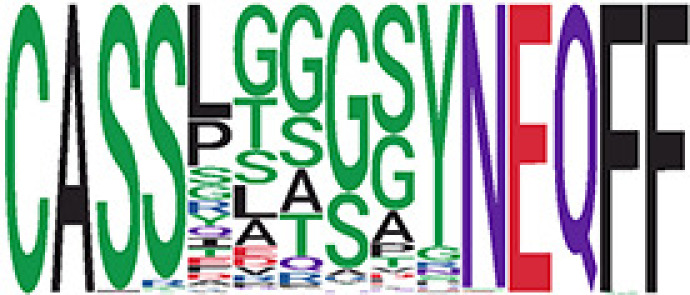	9	34	15	−0.07 (−0.48, 0.34) *p* = 0.733	0.27 (−0.18, 0.73) *p* = 0.241	0.35 (0.01, 0.68) *p* = 0.045	1[1,9]	84.8%	0.6 [0.46,0.74]	29.0%
16	493	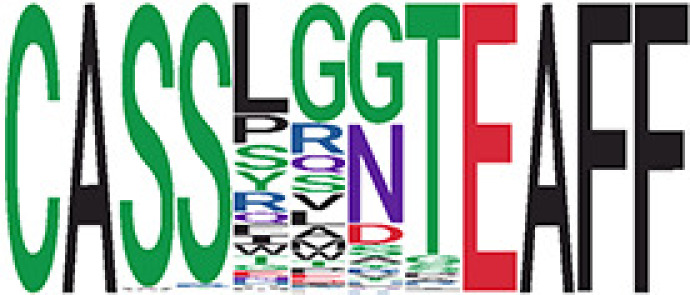	7	13	15	−0.41 (−0.75, −0.07) *p* = 0.017	−0.25 (−0.56, 0.07) *p* = 0.12	0.16 (−0.12, 0.44) *p* = 0.262	1[1,4]	97.0%	0.42 [0.26,0.57]	50.7%
18	681	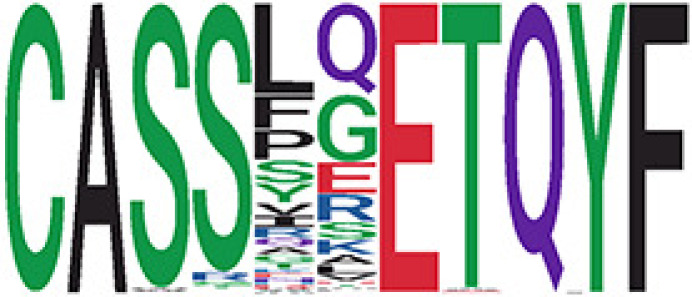	12	27	18	0.13 (−0.14, 0.4) *p* = 0.334	0.34 (0.06, 0.61) *p* = 0.015	0.21 (−0.03, 0.44) *p* = 0.084	3[1,8]	89.7%	0.67 [0.55,0.84]	48.9%
22	698	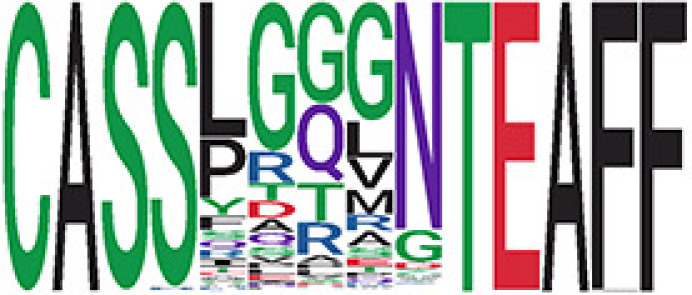	9	16	13	−0.36 (−0.7, −0.02) *p* = 0.036	−0.34 (−0.66, −0.02) *p* = 0.039	−0.25 (−0.79, 0.28) *p* = 0.352	1[1,4]	92.1%	0.41 [0.26,0.56]	28.1%
27	334	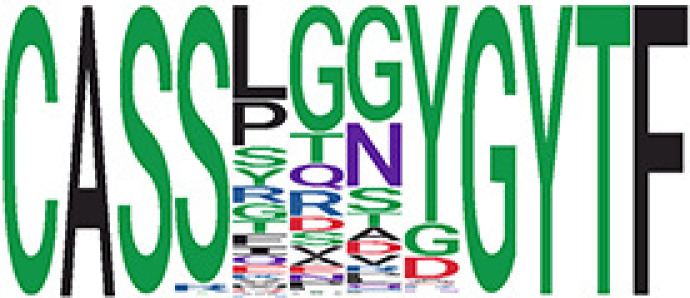	5	12	10	−0.52 (−0.9, −0.13) *p* = 0.008	−0.39 (−0.77, 0) *p* = 0.051	0.13 (−0.18, 0.45) *p* = 0.409	1[1,5]	92.8%	0.47 [0.31,0.62]	25.7%
32	103	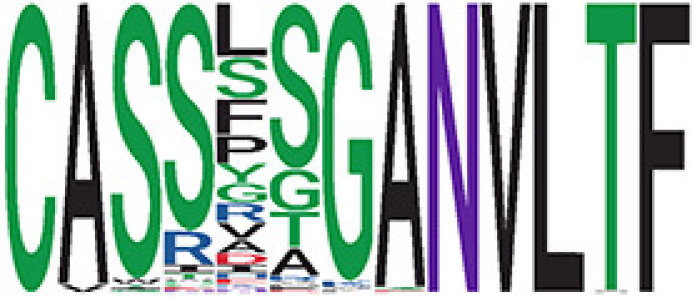	4	9	8	−0.75 (−1.39, −0.1) *p* = 0.023	−1.18 (−1.84, −0.52) *p*< 0.001	−0.43 (−0.95, 0.09) *p* = 0.104	1[1,5]	77.7%	0.35 [0.15,0.55]	35.9%
44	34	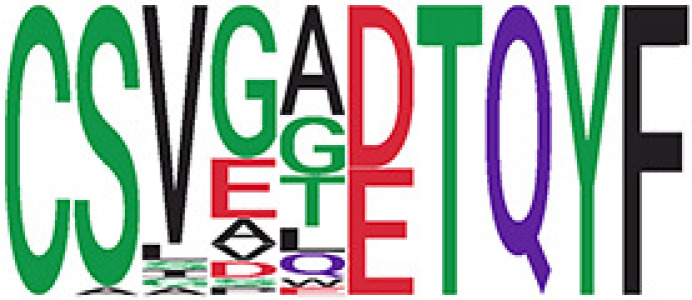	0	8	4	NA	NA	−0.46 (−0.82, -0.1) *p* = 0.012	1[1,1]	52.9%	0.44 [0.29,0.6]	5.9%

^ix^Public cluster ID is defined based on the network analysis across all samples.

^ix^Sequence logo visualizes all TCR sequences within the corresponding public cluster.

^ix^The number of the healthy donor (HD) samples which the corresponding public cluster belongs to.

^ix^The number of the active COVID samples which the corresponding public cluster belongs to.

^ix^The number of the recovered COVID samples which the corresponding public cluster belong to.

^ix^Estimate (with 95% CI) and p-value were obtained by a linear mixed model or a linear model.

^ix^Summary of the coreness (local property) of the TRCs in the corresponding network cluster.

^ix^The percentage of the significant TCRs within each public cluster based on Bayes factor FDR< 0.05.

^ix^Summary statistics [median (Interquartile range, IQR)] of pairwise correlation coefficients between the TCR sequences within the public cluster, where the amino acid sequences were transformed by Atchley factor.

^ix^The percentage of the TCRs in the public cluster matched with MIRA.

Clusters 1, 4, 6, 7, 8, and 18, highlighted in light pink, with positive estimates and p< 0.05 in either the active versus HD or recovered versus HD columns were considered to be associated with COVID-19 samples. Clusters 2, 9, 16, 22, 27, and 32, highlighted in light green, with negative estimates and p< 0.05 in either the active versus HD or recovered versus HD columns, were considered to be associated with HD samples. Similarly, Clusters 1, 2, 8, and 11, highlighted in light pink, with positive estimates and p< 0.05 in recovered versus active columns were therefore considered to be associated with COVID-19–recovered samples, while Cluster 44, highlighted in light green, had negative estimates and p< 0.05 was considered to be associated with COVID-active samples.

### 3.4 Comparing with GLIPH2 results

We further compared our findings with the results obtained by GLIPH2 (**Supplementary Table S5**). Using GLIPH2 to analyze the European COVID datasets, we found 57,943 TCRs within 4,009 patterns when comparing COVID samples *versus* HD samples (*p*< 0.05, out of 833,028 TCRs within 156,383 patterns). Downstream differential testing based on TCR clonal abundance was applied to the 3,979 TCR clusters (32,282 unique TCRs) with at least three position matching. **Supplementary Table S5** presents the number of unique TCR clones identified by both Network Analysis of Immune Repertoire (NAIR) and GLIPH2, their overlaps, and the number of those TCR clones matched with the MIRA database after initial searching and after differential testing. Interestingly, the TCRs identified by both approaches are more likely to be validated by the MIRA database (last column). NAIR consistently outperforms much better than GLIPH2 based on the number of TCRs validated by the MIRA database.

### 3.5 Sensitivity analysis

Because clonal grouping can be impacted by experimental factors such as sampling and sequencing depth, we performed a sensitivity analysis on one active sample and one recovered sample for illustration purposes (**Supplementary Figures S3A, B**). We subsampled clones to achieve similar sequencing depth of 5,000, weighted by the distribution of normalized abundance. Although the number and size of the clusters decreased, the general pattern of the network and matching with the MIRA dataset remained (**Supplementary Figures S3C, D**).

To account for the differences in sequence lengths when using Hamming distance, we expanded all sequences to the maximum length by appending zero to the right side of each sequence, a common way to deal with discrepancies in sequence lengths. However, we acknowledge that this alteration may introduce bias into our analysis. As a sensitivity analysis, we also used the Levenshtein distance metric, which is designed to compare the dissimilarity of TCR sequences of different lengths while accounting for gaps and insertions. Although the Levenstein distance (Python module Levenshtein with distance function) *versus* Hamming distance (Python module SciPy with pdist function) (**Supplementary Figures S3E, F**) were similar in structure and pattern, the threshold of distance might play a significant role. Since the current paper focused on the CDR3 amino acid sequence, as discussed in (31), we used a cutoff of 1. However, for full-length nucleotide TCR sequences, a cutoff of 1 is probably too stringent. Based on the sensitivity analysis with different cutoffs (**Supplementary Table S6A**), we found that, as expected with a higher cutoff (i.e., 2), the number of clusters within the network is smaller and the cluster sizes are relatively larger. Such differences are more noticeable when Levenstein distance is applied. However, the differences are less when applied to CDR3 nucleotide sequence analysis.

We chose fast greedy approach as the clustering approach for network analysis due to the fastest speed. We compared the results of all available methods in igraph (fast greedy, walktrap, eigen, betweenness, and Louvain). All approaches generated similar results for small-to-moderate-size clusters except Leiden (**Supplementary Table S6B**). However, when the data have larger nodes, such as when we perform network analysis to obtain the global membership to obtain public clusters, fast greedy and Louvain provide comparable similar results while other methods break the clusters into small-size memberships.

For public cluster searching, the choice of K is arbitrary. As expected, the larger the K is, the more public clusters will be identified. However, the choice is relatively robust regarding the number of clusters shared by more than five samples. Since the identified TCRs in the clusters will usually be used for further validation by either external data or functional analysis, one can choose a loose criterion to include more candidate TCRs.

### 3.6 Computational environment

With a MacBook Pro (Processor of 2.3 GHz Intel Core i9 and Memory of 32 GB 2400 MHz DDR4), it uses 50–1,700 Mb memory to perform network analysis (Hamming distance) on a sample with 1,000–50,000 clones by using the current version of the software, which takes 2 s to 35 min, respectively. It takes much longer (up to 25 times longer) if Levenshtein distance is applied.

## 4 Conclusion and discussion

Due to the heterogeneous nature of T cells across time and different subjects, analyses of AIRR-seq data have been challenging. Network analysis allows us to potentially uncover the biological significance of unique TCRs based on sequence. We developed two different pipelines to identify disease-specific clusters and public clusters, along with novel metrics to evaluate the identified clusters along with downstream analysis. The workflows can be applied to B-cell repertoires directly and can be easily extended to identify the clusters that respond to treatment (32).

COVID-19 epitope-specific TCR clones in MIRA were also found in the European datasets, suggesting that COVID-19 subjects develop a distinct subset of T cells against just a few epitopes (18). When matching with MIRA data, results indicated a higher proportion of COVID-19–specific TCRs in recovered subjects, implying TCRs might be used as a prognostic marker. Interestingly, samples from subjects with active COVID-19 infection were more similar to those of healthy donors. This is consistent with a previous study that demonstrated that patients who have recovered from COVID-19 had a more diverse repertoire compared with active COVID-19 infection and healthy donors (20). Patients with active COVID-19 infection may not have developed an adequate T-cell response to clear infection. Thus, their repertoire appears similar to healthy donors. It is not surprising that more public TCR clusters were detected within the MIRA dataset but fewer COVID-specific clusters matched with MIRA datasets. As shown in **Table 2**, those public TCR clusters have a relatively high probability of generation, which means they have a higher chance to present in human subjects (including the MIRA dataset). In comparison, the COVID-specific clusters have a relatively low probability of generation (**Table 1**) or a lower chance of presenting in human subjects. The MIRA dataset is still under construction, so it does not have a complete list of COVID-related epitopes. Furthermore, the MIRA dataset mainly collected samples from North America instead of Europe, and it used Adaptive ImmunoSQE (21), while the European data used customized NGS (20).

Several methods for the computational and statistical analysis of large-scale AIRR-seq data have been developed to resolve immune repertoire complexity and to understand the dynamics of adaptive immunity by using network analysis, such as GLIPH2 (18), ImmunoMap (19), TESSA (27), and iSMART (33). However, GLIPH2, iSMART, and TESSA focus more on single-cell RNA sequences. The comparison with GLIPH2 shows that our proposed pipelines can identify more COVID-specific TCRs (**Supplementary Table S5**). Like many other computational approaches, our method involved selecting specific methods and parameters. To assess the robustness of our results to these choices, we conducted brief sensitivity analyses of the critical options (**Supplementary Figure S3**, **Supplementary Table S6**). We found that the results were relative robust to variations in these options and parameters.

One limitation of our proposed method is that we have used a dataset derived from COVID-19 subjects. SARS-CoV-2, the virus responsible for COVID-19, has relatively fewer epitopes compared with more complex diseases, such as malignancies with high-mutational burden (33). Thus, applying the proposed analysis workflow to study the immune response in other diseases, such as cancer and treatment (e.g., cancer immunotherapy), may be significantly more complex. Although the proposed pipeline can be applied to diverse sequence assays, if the objective is to identify public or related clusters, then it is advisable to use TCR sequences obtained from the same sequencing assay. Additionally, another limitation to searching for disease-associated clusters is that the initial implementation of Fisher’s exact test requires an adequate sample sizes. Finally, human leukocyte antigens (HLA) are proteins that help individual immune cells from distinguishing between foreign and self. HLA alleles have been correlated to incidence and severity of diseases such as COVID-19. For example, HLA-B*15:03 has been shown to present a larger array of peptides and individuals with Class I HLA alleles have milder COVID-19 infections compared with other individuals with higher heterozygosity. Interestingly, HLA-B*15:03 is prevalent in West Africa and countries with high-endemic malaria. Our data do not have the complete set of HLA alleles possible, because much of our data come from Europe. A larger and more diverse dataset including HLA allele information combined with identified COVID-specific can provide a more comprehensive understanding of the T-cell response to COVID-19.

In conclusion, we have developed a bioinformatics pipeline by incorporating the proposed methods and techniques to tackle the complexity of the immunosequencing data in a translational fashion. The associations found in our study need further functional studies to confirm the biological significance and to explore their clinical applications. Validation of TCR antigen specificity traditionally require identification of antigen-specific TCRs with peptide/HLA multimers then expression and functional testing of identified TCRs, which is labor and time intensive. This *in-silico* bioinformatic approach can improve the current workflows by narrowing the number of TCRs that need to be tested.

## Data availability statement

Publicly available datasets were analyzed in this study. This data can be found here: The European data are available in gateway.ireceptor.org; Study ID: IR-Binder-000001 *via* The Adaptive Immune Receptor Repertoire (AIRR) Data Commons. Availability and Implementation The code and scripts have been deposited in GitHub (https://github.com/mlizhangx/Network-Analysis-for-Repertoire-Sequencing-).

## Ethics statement

Ethical review and approval was not required for the study on human participants in accordance with the local legislation and institutional requirements. The patients/participants provided their written informed consent to participate in this study.

## Author contributions

HY, JC and LZ initiated and designed the experiment. HY, ZF and LZ acquired the data. YH, ZF, TH and LZ performed the data analysis. HY and BN developed the software. TH and LZ acquired the funding. All authors contributed to the article and approved the submitted version.

## Supplementary material

Supplementary Figure 1 Network properties. **(A)** Boxplot of the (log_10_ transformed) number of the clusters within each sample. **(B)** Heatmap of the number of the clusters within each sample. Each row represents an individual sample (with left bar presenting the sample information), while the column corresponds to the number of the clusters. **(C)** Boxplot of the (log_10_ transformed) maximum number of the T-cell receptors (TCRs) within each of the clusters for each sample. **(D)** Heatmap of the number of the TCRs within each of the clusters for each sample. Each row represents an individual sample (with left bar presenting the sample information), while each column corresponds to the number of the TCRs within each of the clusters for each sample. **(E)** Boxplot of the maximum assortativity within each of the clusters of the clusters for each sample. **(F)** Heatmap of the assortativity within each of the clusters of the clusters for each sample. Each row represents an individual sample (with left bar presenting the sample information), while each column corresponds to the assortativity within each of the clusters of the clusters for each sample.

Supplementary Figure 2 Representative COVID-only TCR clusters. **(A–C)** Each figure presents a T-cell receptor (TCR) cluster with the motif showing on the top. Each node represents a single TCR which are connected if the distance between the two nodes is<= 1, with node size is proportional to the TCR clonal abundance. The color code in the top panel presents if the TCRs belong to active (red) or recovered (blue) samples, while in the bottom panel, color corresponds to each individual subject information.

Supplementary Figure 3 Sensitive analysis. **(A, B)** Network analysis of samples Pt-5-2 (actives) and Pt-7-4 (recovered) based on the original data using Hamming distance. **(C, D)** Network analysis of samples Pt-5-2 (actives) and Pt-7-4 (recovered) based on the subsampling using Hamming distance. **(E, F)** Network analysis of samples Pt-5-2 (actives) and Pt-7-4 (recovered) based on the original data using Levenshtein distance. In each figure, each node represents a single TCR which are connected if the distance between the two nodes is<= 1. Colored dots represent if matched with MIRA data (green) or not (red).

## References

[1] Miho E Yermanos A Weber CR Berger CT Reddy ST Greiff V . Computational strategies for dissecting the high-dimensional complexity of adaptive immune repertoires. Front Immunol (2018) 9:224. doi: 10.3389/fimmu.2018.00224 29515569 PMC5826328

[2] Hammarlund E Lewis MW Carter SV Amanna I Hansen SG Strelow LI . Multiple diagnostic techniques identify previously vaccinated individuals with protective immunity against monkeypox. Nat Med (2005) 11:1005–11. doi: 10.1038/nm1273 16086024

[3] Amanna IJ Carlson NE Slifka MK . Duration of humoral immunity to common viral and vaccine antigens. N Engl J Med (2007) 357:1903–15. doi: 10.1056/NEJMoa066092 17989383

[4] Manz RA Thiel A Radbruch A . Lifetime of plasma cells in the bone marrow. Nature (1997) 388:133–4. doi: 10.1038/40540 9217150

[5] Landsverk OJB Snir O Casado RB Richter L Mold JE Réu P . Antibody-secreting plasma cells persist for decades in human intestine. J Exp Med (2017) 214(2):309–17. doi: 10.1084/jem.20161590 PMC5294861 28104812

[6] Halliley JL Tipton CM Liesveld J Rosenberg AF Darce J Gregoretti IV . Long-lived plasma cells are contained within the CD19–CD38hiCD138+ subset in human bone marrow. Immunity (2015) 43(1):132–45. doi: 10.1016/j.immuni.2015.06.016 PMC4680845 26187412

[7] Pollok K Mothes R Ulbricht C Liebheit A Gerken JD Uhlmann S . The chronically inflamed central nervous system provides niches for long- lived plasma cells. Acta Neuropathol Commun (2017) 5:88. doi: 10.1186/s40478-017-0487-8 29178933 PMC5702095

[8] Wang P Jin X Zhou W Luo M Xu Z Xu C . Comprehensive analysis of TCR repertoire in COVID-19 using single cell sequencing. Genomics (2021) 113(2):456–62. doi: 10.1016/j.ygeno.2020.12.036 PMC7833309 33383142

[9] Jin X Zhou W Luo M Wang P Xu Z Ma K . Global characterization of b cell receptor repertoire in COVID-19 patients by single-cell V(D)J sequencing. Brief Bioinform (2021) 22(6):bbab192. doi: 10.1093/bib/bbab192 34015809 PMC8194558

[10] Kotagiri P Mescia F Rae WM Bergamaschi L Tuong ZK Turner L . B cell receptor repertoire kinetics after SARS-CoV-2 infection and vaccination. Cell Rep (2022) 38(7):110393. doi: 10.1016/j.celrep.2022.110393 35143756 PMC8801326

[11] Zhang L Cham J Paciorek A Trager J Sheikh N Fong L . 3D: diversity, dynamics, differential testing – a proposed pipeline for analysis of next-generation sequencing T cell repertoire data. BMC Bioinf (2017) 18:129. doi: 10.1186/s12859-017-1544-9 PMC5327583 28241742

[12] Bashford-Rogers RJM Palser AL Huntly BJ ance R Vassiliou GS Follows GA . Network properties derived from deep sequencing of human b-cell receptor repertoires delineate b-cell populations. Genome Res (2013) 23:1874–84. doi: 10.1101/gr.154815.113 PMC3814887 23742949

[13] Miho E Roškar R Greiff V Reddy ST . Large-Scale network analysis reveals the sequence space architecture of antibody repertoires. Nat Commun (2019) 10:1321. doi: 10.1038/s41467-019-09278-8 30899025 PMC6428871

[14] Robins HS Campregher PV Srivastava SK Wacher A Turtle CJ Kahsai O . Comprehensive assessment of T-cell receptor beta-chain diversity in alphabeta T cells. Proc Natl Acad Sci (2009) 106(10):4144–9. doi: 10.1073/pnas.0813101106 PMC2774550 19706884

[15] Madi A Shifrut E Reich-Zeliger S Gal H Best K Ndifon W . T-Cell receptor repertoires share a restricted set of public and abundant CDR3 sequences that are associated with self-related immunity. Genome Res (2014) 24:1603–12. doi: 10.1101/gr.170753.113 PMC4199372 25024161

[16] Sethna Z Isacchini G Dupic T Mora T Walczak AM Elhanati Y . Population variability in the generation and selection of T-cell repertoires. PloS Comput Biol (2020) 16(12):e1008394. doi: 10.1371/journal.pcbi.1008394 33296360 PMC7725366

[17] Murugan A Mora T Walczak AM Callan CG . Statistical inference of the generation probability of T-cell receptors from sequence repertoires. Proc Natl Acad Sci USA (2012) 109(40):16161–6. doi: 10.1073/pnas.1212755109 PMC3479580 22988065

[18] Huang H Wang C Rubelt F Scriba TJ Davis MM . Analyzing the mycobacterium tuberculosis immune response by T-cell receptor clustering with GLIPH2 and genome-wide antigen screening. Nat Biotechnol (2020) 38(10):1194–202. doi: 10.1038/s41587-020-0505-4 PMC7541396 32341563

[19] Sidhom JW Bessell CA Havel JJ Kosmides A Chan TA Schneck JP . ImmunoMap: a bioinformatics tool for T-cell repertoire analysis. Cancer Immunol Res (2018) 6(2):151–62. doi: 10.1158/2326-6066.CIR-17-0114 PMC6478441 29263161

[20] Schultheiß C Paschold L Simnica D Mohme M Willscher E von Wenserski L . Next-generation sequencing of T and b cell receptor repertoires from COVID-19 patients showed signatures associated with severity of disease. Immunity (2020) 53(2):442–55. doi: 10.1016/j.immuni.2020.06.024 PMC7324317 32668194

[21] Nolan S Vignali M Klinger M Dines JN Kaplan IM Svejnoha E . A large-scale database of T-cell receptor beta (TCRβ) sequences and binding associations from natural and synthetic exposure to SARS-CoV-2. Res Sq [Preprint] (2020) 4:rs.3.rs–51964. doi: 10.21203/rs.3.rs-51964/v1 PMC11873104 40034696

[22] Scott C Ademar A George B Breden F Bukhari SAC Busse CE . The ADC API: a web API for the programmatic query of the AIRR data commons. Front Big Data (2020) 3:22. doi: 10.3389/fdata.2020.00022 33693395 PMC7931935

[23] Bolotin D Poslavsky S Mitrophanov I Shugay M Mamedov IZ Putintseva EV . MiXCR: software for comprehensive adaptive immunity profiling. Nat Methods (2015) 12:380–1. doi: 10.1038/nmeth.3364 25924071

[24] Csárdi G Nepusz T Traag V Horvát S Zanini F Noom D Müller K . igraph: Network Analysis and Visualization in R. doi:10.5281/zenodo.7682609, R package version 1.5.0 (2023). Available at: https://CRAN.R-project.org/package=igraph.

[25] Wagih O . Ggseqlogo: a versatile r package for drawing sequence logos. (2017). doi: 10.1093/bioinformatics/btx469 29036507

[26] Atchley WR Zhao J Fernandes AD Drüke T . Solving the protein sequence metric problem. Proc Natl Acad Sci U.S.A. (2005) 102(18):6395–400. doi: 10.1073/pnas.0408677102 PMC1088356 15851683

[27] Zhang Z Xiong D Wang X Liu H Wang T . Mapping the functional landscape of T cell receptor repertoires by single-T cell transcriptomics. Nat Methods (2021) 18(1):92–9. doi: 10.1038/s41592-020-01020-3 PMC7799492 33408405

[28] Sethna Z Elhanati Y Callan CG Walczak AM Mora T . OLGA: fast computation of generation probabilities of b- and T-cell receptor amino acid sequences and motifs. Bioinformatics (2019) 35(17):2974–81. doi: 10.1093/bioinformatics/btz035 PMC6735909 30657870

[29] Kass RE Raftery AE . Bayes factors. J Am Stat Assoc (1995) 90(430):773–95. doi: 10.1080/01621459.1995.10476572

[30] Benjamini Y Hochberg Y . Controlling the false discovery rate: a practical and powerful approach to multiple testing. J R Stat Soc Ser B (1995) 57:289–300. doi: 10.1111/j.2517-6161.1995.tb02031.x

[31] Meysman P De Neuter N Van de Sande B Ruyssinck J Ronsse M De Baets G . On the viability of unsupervised T-cell receptor sequence clustering for epitope preference. BMC Bioinf (2018) 19(1):85. doi: 10.1186/s12859-018-2085-5 30247624

[32] Zhang L Kandadi H Yang H Cham J He T Oh DY . Long-term sculpting of the b-cell repertoire following cancer immunotherapy in patients treated with sipuleucel-T. Cancer Immunol Res (2020) 8(12):1496–507. doi: 10.1158/2326-6066.CIR-20-0308 PMC7903967 32967912

[33] Zhang H Liu L Zhang J Chen J Ye J Shukla S . Investigation of antigen-specific T-cell receptor clusters in human cancers. Clin Cancer Res (2020) 26(6):1359–71. doi: 10.1158/1078-0432.CCR-19-3249 31831563

