# Supplementary figures and images for: NAIR: Network Analysis of Immune Repertoire
Source: Front Immunol. 2023 Jul 7;14:1181825. doi: 10.3389/fimmu.2023.1181825 (PMC10443597; doi:10.3389/fimmu.2023.1181825)

### Supplementary Figure 1

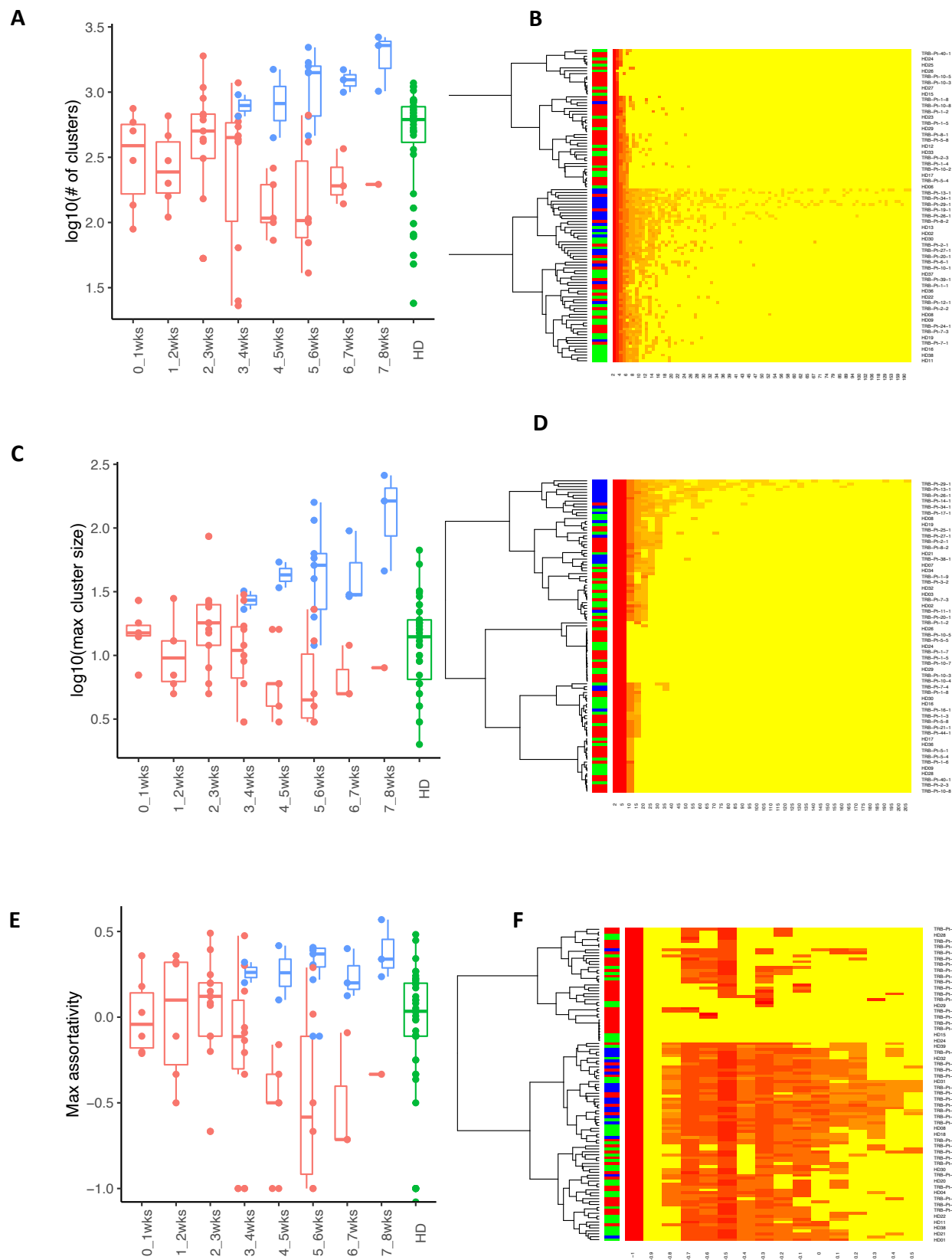

Supplement: Supplementary Figure 1 — Network properties. (A) Boxplot of the (log10 transformed) number of the clusters within each sample. (B) Heatmap of the number of the clusters within each sample. Each row represents an individual sample (with left bar presenting the sample information), while the column corresponds to the number of the clusters. (C) Boxplot of the (log10 transformed) maximum number of the T-cell receptors (TCRs) within each of the clusters for each sample. (D) Heatmap of the number of the TCRs within each of the clusters for each sample. Each row represents an individual sample (with left bar presenting the sample information), while each column corresponds to the number of the TCRs within each of the clusters for each sample. (E) Boxplot of the maximum assortativity within each of the clusters of the clusters for each sample. (F) Heatmap of the assortativity within each of the clusters of the clusters for each sample. Each row represents an individual sample (with left bar presenting the sample information), while each column corresponds to the assortativity within each of the clusters of the clusters for each sample. [file Image_1.pdf]

Supplementary Figure 2

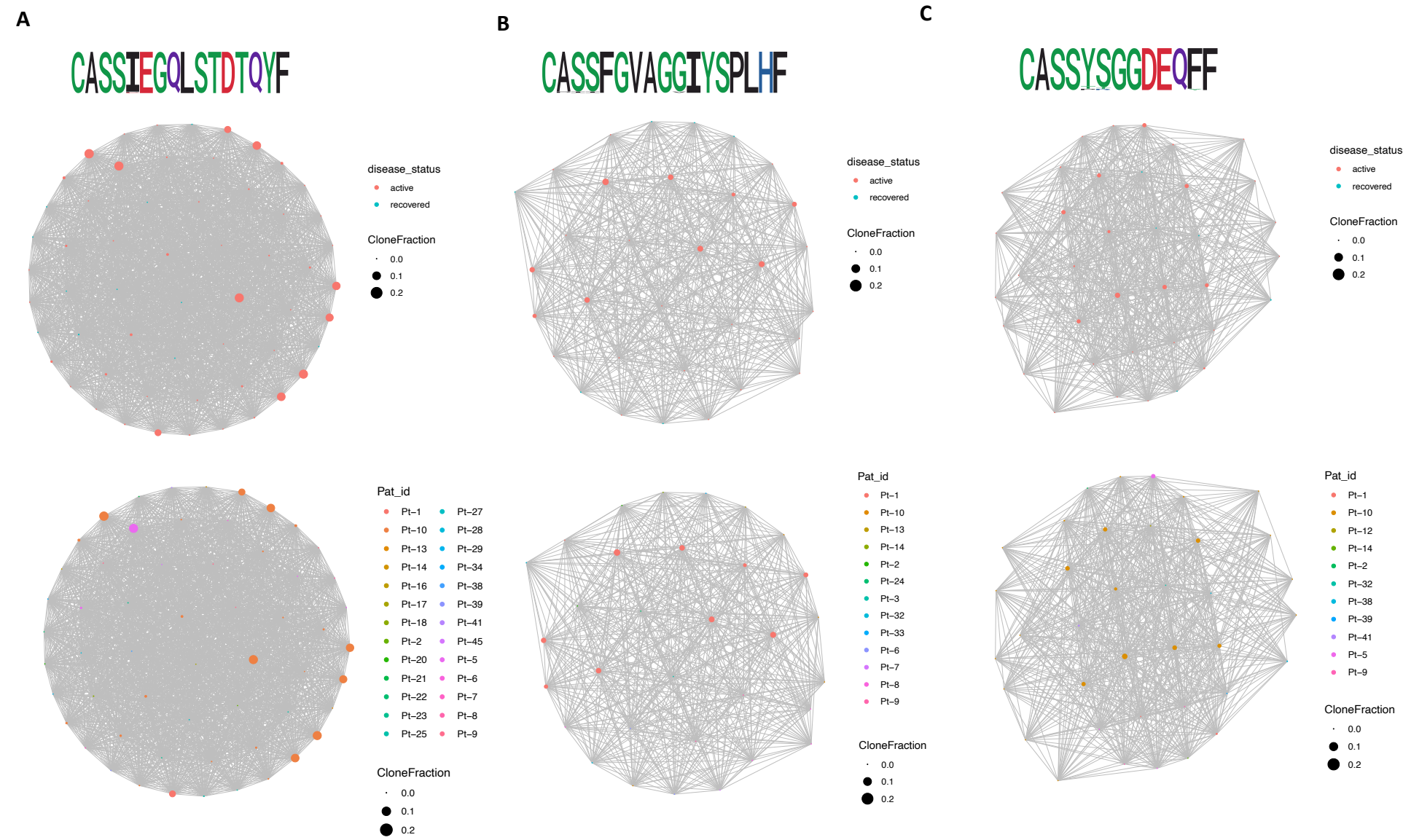

Supplement: Supplementary Figure 2 — Representative COVID-only TCR clusters. (A–C) Each figure presents a T-cell receptor (TCR) cluster with the motif showing on the top. Each node represents a single TCR which are connected if the distance between the two nodes is<= 1, with node size is proportional to the TCR clonal abundance. The color code in the top panel presents if the TCRs belong to active (red) or recovered (blue) samples, while in the bottom panel, color corresponds to each individual subject information. [file Image_2.pdf]

Supplementary Figure 3

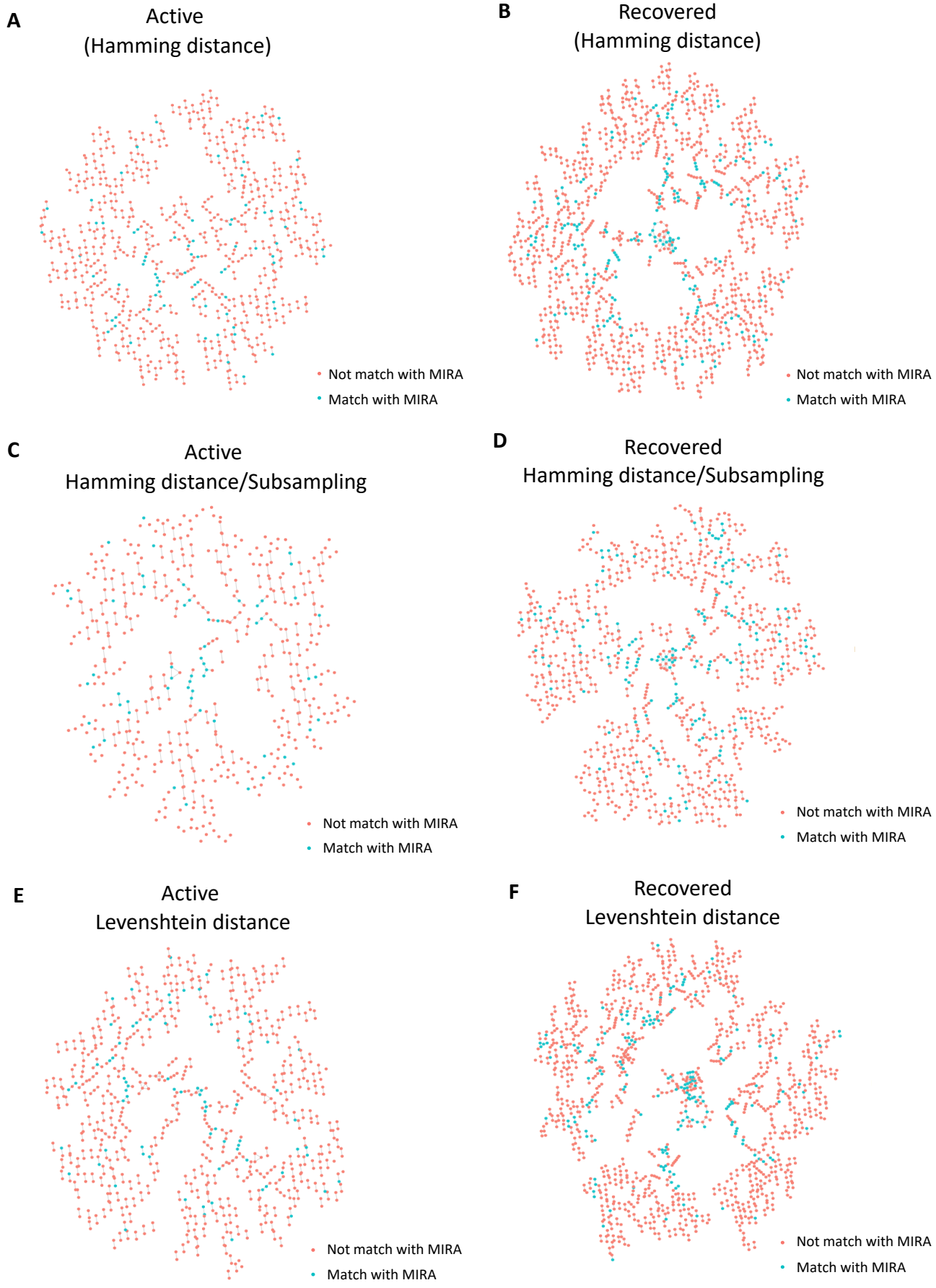

Supplement: Supplementary Figure 3 — Sensitive analysis. (A, B) Network analysis of samples Pt-5-2 (actives) and Pt-7-4 (recovered) based on the original data using Hamming distance. (C, D) Network analysis of samples Pt-5-2 (actives) and Pt-7-4 (recovered) based on the subsampling using Hamming distance. (E, F) Network analysis of samples Pt-5-2 (actives) and Pt-7-4 (recovered) based on the original data using Levenshtein distance. In each figure, each node represents a single TCR which are connected if the distance between the two nodes is<= 1. Colored dots represent if matched with MIRA data (green) or not (red). [file Image_3.pdf]
