# Supplementary material for: NAIR: Network Analysis of Immune Repertoire
Source: Front Immunol. 2023 Jul 7;14:1181825. doi: 10.3389/fimmu.2023.1181825 (PMC10443597; doi:10.3389/fimmu.2023.1181825)
Supplement: Supplementary file 4 [file Table_1.docx]

**Supplementary Table 1**: Summary of the samples

| **Patient ID** | **Sample ID** | **Disease Stage** | **Days from first symptoms** | **Weeks from first symptoms** | **Status/Treatment** |
| --- | --- | --- | --- | --- | --- |
| 1 | 1_1 | active | 2 | 0_1wks | ventilation |
| 1 | 1_2 | active | 6 | 0_1wks | ventilation |
| 1 | 1_3 | active | 8 | 1_2wks | ventilation |
| 1 | 1_4 | active | 14 | 2_3wks | ventilation |
| 1 | 1_5 | active | 16 | 2_3wks | ventilation |
| 1 | 1_6 | active | 20 | 2_3wks | ventilation |
| 1 | 1_7 | active | 23 | 3_4wks | ECMO |
| 1 | 1_8 | active | 27 | 3_4wks | ECMO |
| 1 | 1_9 | active | 29 | 4_5wks | Death |
| 2 | 2_1 | active | 5 | 0_1wks | symptomatic |
| 2 | 2_2 | active | 9 | 1_2wks | ventilation |
| 2 | 2_3 | active | 11 | 1_2wks | ECMO |
| 3 | 3_1 | active | 1 | 0_1wks | symptomatic |
| 3 | 3_2 | active | 5 | 0_1wks | ventilation |
| 5 | 5_1 | active | 20 | 2_3wks | ECMO |
| 5 | 5_2 | active | 26 | 3_4wks | ECMO |
| 5 | 5_3 | active | 28 | 4_5wks | ECMO |
| 5 | 5_4 | active | 32 | 4_5wks | ECMO |
| 5 | 5_5 | active | 35 | 5_6wks | ECMO |
| 5 | 5_6 | active | 39 | 5_6wks | ECMO |
| 5 | 5_7 | active | 41 | 5_6wks | ECMO |
| 5 | 5_8 | active | 46 | 6_7wks | ECMO |
| 6 | 6_1 | active | 18 | 2_3wks | symptomatic |
| 6 | 6_2 | recovered | 23 | 3_4wks | recovered |
| 7 | 7_1 | active | 15 | 2_3wks | symptomatic |
| 7 | 7_2 | active | 20 | 2_3wks | symptomatic |
| 7 | 7_3 | active | 23 | 3_4wks | symptomatic |
| 7 | 7_4 | recovered | 27 | 3_4wks | recovered |
| 8 | 8_1 | active | 18 | 2_3wks | ECMO |
| 8 | 8_2 | active | 24 | 3_4wks | ECMO |
| 9 | 9_1 | active | 16 | 2_3wks | ECMO |
| 9 | 9_2 | active | 22 | 3_4wks | ECMO |
| 10 | 10_1 | active | 25 | 3_4wks | ECMO |
| 10 | 10_2 | active | 31 | 4_5wks | ECMO |
| 10 | 10_3 | active | 33 | 4_5wks | ECMO |
| 10 | 10_4 | active | 37 | 5_6wks | ECMO |
| 10 | 10_5 | active | 40 | 5_6wks | ECMO |
| 10 | 10_6 | active | 44 | 6_7wks | ECMO |
| 10 | 10_7 | active | 46 | 6_7wks | ECMO |
| 10 | 10_8 | active | 51 | 7_8wks | ECMO |
| 11 | 11_1 | recovered | 37 | 5_6wks | recovered |
| 12 | 12_1 | recovered | 31 | 4_5wks | recovered |
| 13 | 13_1 | recovered | 36 | 5_6wks | recovered |
| 14 | 14_1 | recovered | 38 | 5_6wks | recovered |
| 16 | 16_1 | recovered | 38 | 5_6wks | recovered |
| 17 | 17_1 | recovered | 39 | 5_6wks | recovered |
| 18 | 18_1 | recovered | 39 | 5_6wks | recovered |
| 19 | 19_1 | active | 9 | 1_2wks | symptomatic |
| 20 | 20_1 | active | 24 | 3_4wks | symptomatic |
| 21 | 21_1 | active | 20 | 2_3wks | symptomatic |
| 22 | 22_1 | active | 40 | 5_6wks | ventilation |
| 23 | 23_1 | active | 23 | 3_4wks | symptomatic |
| 24 | 24_1 | active | 20 | 2_3wks | symptomatic |
| 25 | 25_1 | active | 16 | 2_3wks | ECMO |
| 25 | 25_2 | active | 21 | 3_4wks | ECMO |
| 26 | 26_1 | recovered | 45 | 6_7wks | recovered |
| 27 | 27_1 | recovered | 45 | 6_7wks | recovered |
| 28 | 28_1 | recovered | 52 | 7_8wks | recovered |
| 29 | 29_1 | recovered | 51 | 7_8wks | recovered |
| 32 | 32_1 | recovered | 34 | 4_5wks | recovered |
| 33 | 33_1 | recovered | 39 | 5_6wks | recovered |
| 34 | 34_1 | recovered | 39 | 5_6wks | recovered |
| 35 | 35_1 | recovered | 47 | 6_7wks | recovered |
| 38 | 38_1 | recovered | 50 | 7_8wks | recovered |
| 39 | 39_1 | active | 1 | 0_1wks | symptomatic |
| 40 | 40_1 | active | 13 | 1_2wks | ventilation |
| 41 | 41_1 | active | 11 | 1_2wks | ventilation |
| 44 | 44_1 | active | 15 | 2_3wks | ECMO |
| 45 | 45_1 | recovered | 37 | 5_6wks | recovered |
| HD01 | HD01 | healthy |  |  |  |
| HD02 | HD02 | healthy |  |  |  |
| HD03 | HD03 | healthy |  |  |  |
| HD04 | HD04 | healthy |  |  |  |
| HD05 | HD05 | healthy |  |  |  |
| HD06 | HD06 | healthy |  |  |  |
| HD07 | HD07 | healthy |  |  |  |
| HD08 | HD08 | healthy |  |  |  |
| HD09 | HD09 | healthy |  |  |  |
| HD10 | HD10 | healthy |  |  |  |
| HD11 | HD11 | healthy |  |  |  |
| HD12 | HD12 | healthy |  |  |  |
| HD13 | HD13 | healthy |  |  |  |
| HD14 | HD14 | healthy |  |  |  |
| HD15 | HD15 | healthy |  |  |  |
| HD16 | HD16 | healthy |  |  |  |
| HD17 | HD17 | healthy |  |  |  |
| HD18 | HD18 | healthy |  |  |  |
| HD19 | HD19 | healthy |  |  |  |
| HD20 | HD20 | healthy |  |  |  |
| HD21 | HD21 | healthy |  |  |  |
| HD22 | HD22 | healthy |  |  |  |
| HD23 | HD23 | healthy |  |  |  |
| HD24 | HD24 | healthy |  |  |  |
| HD25 | HD25 | healthy |  |  |  |
| HD26 | HD26 | healthy |  |  |  |
| HD27 | HD27 | healthy |  |  |  |
| HD28 | HD28 | healthy |  |  |  |
| HD29 | HD29 | healthy |  |  |  |
| HD30 | HD30 | healthy |  |  |  |
| HD31 | HD31 | healthy |  |  |  |
| HD32 | HD32 | healthy |  |  |  |
| HD33 | HD33 | healthy |  |  |  |
| HD34 | HD34 | healthy |  |  |  |
| HD35 | HD35 | healthy |  |  |  |
| HD36 | HD36 | healthy |  |  |  |
| HD37 | HD37 | healthy |  |  |  |
| HD38 | HD38 | healthy |  |  |  |
| HD39 | HD39 | healthy |  |  |  |
