# Supplementary material for: NAIR: Network Analysis of Immune Repertoire
Source: Front Immunol. 2023 Jul 7;14:1181825. doi: 10.3389/fimmu.2023.1181825 (PMC10443597; doi:10.3389/fimmu.2023.1181825)
Supplement: Supplementary file 6 [file Table_3.docx]

**Supplementary Table 3:** Network Properties Comparisons across Different Subject Groups

| **Network Property** | **Comparison** | **Estimate (95% CI, pvalue)^1^** |
| --- | --- | --- |
| log_10_(# of clusters) | active vs. healthy | -0.1 ( -0.3 , 0.1 p= 0.332 ) |
| **log**_10_**( # of clusters)** | **recovered vs. active** | **0.67 ( 0.39 , 0.95 p < 0.001 )** |
| **log**_10_**( # of clusters)** | **recovered vs. healthy** | **0.38 ( 0.16 , 0.6 p= 0.001 )** |
| log_10_(max cluster size) | active vs. healthy | -0.02 ( -0.18 , 0.14 p= 0.786 ) |
| **log**_10_**(max cluster size)** | **recovered vs. active** | **0.8 ( 0.6 , 1.01 p < 0.001 )** |
| **log**_10_**(max cluster size)** | **recovered vs. healthy** | **0.59 ( 0.4 , 0.77 p < 0.001 )** |
| Max diameter | active vs. healthy | -0.18 ( -1.81 , 1.45 p= 0.827 ) |
| **Max diameter** | **recovered vs. active** | **7.36 ( 5.15 , 9.56 p < 0.001 )** |
| **Max diameter** | **recovered vs. healthy** | **5.27 ( 3.41 , 7.14 p < 0.001 )** |
| Max assortativity | active vs. healthy | -0.08 ( -0.25 , 0.08 p= 0.327 ) |
| **Max assortativity** | **recovered vs. active** | **0.7 ( 0.48 , 0.92 p < 0.001 )** |
| **Max assortativity** | **recovered vs. healthy** | **0.33 ( 0.13 , 0.53 p= 0.001 )** |

Estimate (95% CI, pvalue)^1^ was obtained by generalized linear mixed model. CI stands for confidence interval of the coefficient estimate.
