# Supplementary material for: NAIR: Network Analysis of Immune Repertoire
Source: Front Immunol. 2023 Jul 7;14:1181825. doi: 10.3389/fimmu.2023.1181825 (PMC10443597; doi:10.3389/fimmu.2023.1181825)
Supplement: Supplementary file 8 [file Table_5.docx]

**Supplementary Table 5:** Summary of comparisons between NAIR and GLIPH2

|  | **NAIR** | | | **GLIPH2** | | **Overlap between NAIR and GLIPH2** | |
| --- | --- | --- | --- | --- | --- | --- | --- |
|  |  | **No. of unique TCRs** | **No. of TCRs matched with MIRA (%)** | **No. of unique TCRs** | **No. of TCRs matched with MIRA (%)** | **No. of unique TCRs** | **No. of TCRs matched with MIRA (%)** |
| **Initial Searching** | **Public** | 22,276 | 4,241 (19.0%) | 32,282 | 3,055 (9.5%) | 3,879 | 1,102 (28.4%) |
|  | **Disease-associated** | 3,109 | 1,358 (43.7%) | 32,282 | 3,055 (9.5%) | 675 | 350 (51.9%) |
|  | **All** | 23,025 | 4,440 (19.3%) | 32,282 | 3,055 (9.5%) | 4,016 | 1,146 (28.5%) |
| **Significant based on Differential Testing** | **Public** | 9,993 | 2,593 (25.9%) | 5,056 | 547 (13.1%) | 461 | 202 (43.8%) |
|  | **Disease-associated** | 2,821 | 1,300 (46.1%) | 5,056 | 547 (13.1%) | 172 | 109 (63.4%) |
|  | **All** | 11,515 | 3,131 (27.2%) | 5,056 | 547 (13.1%) | 559 | 229 (45.0%) |
