# Supplementary material for: NAIR: Network Analysis of Immune Repertoire
Source: Front Immunol. 2023 Jul 7;14:1181825. doi: 10.3389/fimmu.2023.1181825 (PMC10443597; doi:10.3389/fimmu.2023.1181825)
Supplement: Supplementary file 9 [file Table_6.docx]

**Supplementary Table 6A:** Summary of sensitivity analysis of different distance cutoff for network analysis.

| Sequence Types | Sample ID | Distance Method | Cutoff | Number of clusters (cluster size >=2) | Cluster size  Median (range) |
| --- | --- | --- | --- | --- | --- |
| Amino acid sequence | 7-4 | Hamming | 1 | 652 | 2(2:32) |
|  |  | Hamming | 2 | 507 | 2(2:78) |
|  |  | Levenstein | 1 | 619 | 2(2:42) |
|  |  | Levenstein | 2 | 401 | 2(2:156) |
|  | 5-2 | Hamming | 1 | 463 | 2(2:6) |
|  |  | Hamming | 2 | 423 | 2(2:18) |
|  |  | Levenstein | 1 | 453 | 2(2:8) |
|  |  | Levenstein | 2 | 341 | 2(2:52) |
| Nucleotide  sequence | 7-4 | Hamming | 1 | 1156 | 2(2:174) |
|  |  | Hamming | 2 | 1309 | 2(2:214) |
|  |  | Levenstein | 1 | 1189 | 2(2:181) |
|  |  | Levenstein | 2 | 1354 | 2(2:223) |
|  | 5-2 | Hamming | 1 | 946 | 2(2:107) |
|  |  | Hamming | 2 | 1102 | 2(2:122) |
|  |  | Levenstein | 1 | 982 | 2(2:110) |
|  |  | Levenstein | 2 | 1173 | 2(2:129) |

**Supplementary Table 6B:** Summary of sensitivity analysis of various clustering approaches for network analysis.

| Sample ID | Clustering  Method | Number of clusters (cluster size >=2) | Cluster size  Median (range) |
| --- | --- | --- | --- |
| 7-4 | Fast greedy | 498 | 2(2:30) |
|  | Walktrap | 498 | 2(2:30) |
|  | Eigen | 498 | 2(2:30) |
|  | Betweenness | 498 | 2(2:30) |
|  | Louvain | 498 | 2(2:30) |
|  | Leiden | 656 | 2(1:7) |
| 5-2 | Fast greedy | 351 | 2(2:6) |
|  | Walktrap | 351 | 2(2:6) |
|  | Eigen | 351 | 2(2:6) |
|  | Betweenness | 351 | 2(2:6) |
|  | Louvain | 351 | 2(2:6) |
|  | Leiden | 422 | 2(1:4) |
